# Social network‐based approaches to HIV testing: a systematic review and meta‐analysis

**DOI:** 10.1002/jia2.26353

**Published:** 2024-09-09

**Authors:** Annabelle Choong, Yi Ming Lyu, Cheryl C. Johnson, Rachel Baggaley, Magdalena Barr‐DiChiara, Muhammad S. Jamil, Nandi L. Siegfried, Christopher K. Fairley, Eric P. F. Chow, Virginia Macdonald, Jason J. Ong

**Affiliations:** ^1^ Central Clinical School Monash University Melbourne Victoria Australia; ^2^ Melbourne Sexual Health Centre Alfred Health Melbourne Victoria Australia; ^3^ Global HIV, Hepatitis and STIs Programmes World Health Organization Geneva Switzerland; ^4^ Regional Office to the Eastern Mediterranean World Health Organization Cairo Egypt; ^5^ Independent Clinical Epidemiologist Cape Town South Africa; ^6^ Centre for Epidemiology and Biostatistics, Melbourne School of Population and Global Health The University of Melbourne Melbourne Victoria Australia; ^7^ Faculty of Infectious and Tropical Diseases London School of Hygiene and Tropical Medicine London UK

**Keywords:** HIV, key populations, social network‐based testing, systematic review, test promoters, testing

## Abstract

**Introduction:**

Social network‐based testing approaches (SNAs) encourage individuals (“test promoters”) to motivate sexual partners and/or those in their social networks to test for HIV. We conducted a systematic review to examine the effectiveness, acceptability and cost‐effectiveness of SNA.

**Methods:**

We searched five databases from January 2010 to May 2023, and included studies that compared SNA with non‐SNA. We used random‐effects meta‐analysis to combine effect estimates. Certainty was assessed using the GRADE approach.

**Results:**

We identified 47 studies. SNA may increase uptake of HIV testing compared to non‐SNA (RR 2.04, 95% CI: 1.06–3.95, Low certainty). The proportion of first‐time testers was probably higher among partners or social contacts of test promoters using SNA compared to non‐SNA (RR 1.49, 95% CI: 1.22–1.81, Moderate certainty). The proportion of people who tested positive for HIV may be higher among partners or social contacts of test promoters using SNA compared to non‐SNA (RR 1.84, 95% CI: 1.01–3.35, Low certainty). There were no reports of any adverse events or harms associated with SNA. Based on six cost‐effectiveness studies, SNA was generally cheaper per person tested and per person diagnosed compared to non‐SNA. Based on 23 qualitative studies, SNA is likely to be acceptable to a variety of populations.

**Discussion:**

Our review collated evidence for SNA to HIV testing covering the key populations and the general population who may benefit from HIV testing. We summarized evidence for the effectiveness, acceptability and cost‐effectiveness of different models of SNA. While we did not identify an ideal model of SNA that could be immediately scaled up, for each setting and population targeted, we recommend various implementation considerations as our meta‐analysis showed the effectiveness might differ due to factors which include the testing modality (i.e. use of HIV self‐testing), type of test promoters, long or short duration of recruitment and use of financial incentives.

**Conclusions:**

Social network‐based approaches may enhance HIV testing uptake, increase the proportion of first‐time testers and those testing positive for HIV. Heterogeneity among studies highlights the need for context‐specific adaptations, but the overall positive impact of SNA on HIV testing outcomes could support its integration into existing HIV testing services.

## 1 INTRODUCTION

HIV testing services (HTS) are needed to know one's HIV status and receive appropriate onward prevention and treatment services. While there has been remarkable progress and rollout of HTS, gaps remain. At the global level, the 90‐90‐90 targets for 2020 were missed, and we are not on track to achieve the 95‐95‐95 goals by 2025 [1]. It is estimated that 5.9 million people living with HIV did not know their HIV‐positive status in 2021 [2]. Strategies are thus needed to increase HTS uptake worldwide. The World Health Organization (WHO) recommends countries offer a strategic mix of testing approaches that can be adapted for specific populations. This includes community and facility‐based services, HIV self‐testing (HIVST) and voluntary HIV partner services, including partner notification [3, 4].

WHO has prioritized reaching certain populations to achieve global HIV targets, including key populations, partners of people with HIV globally, as well as adult men, children and adolescent girls, and young women in Eastern and Southern Africa. Key populations are defined by WHO as men who have sex with men (MSM), people who inject drugs (PWID), people who engage in sex work, transgender people and people in prisons and other closed settings. Despite key populations and their sexual partners contributing to the majority of new HIV acquisitions, they largely remain underserved and are less likely to know their status or be on treatment than their peers [1, 5]. Where uptake of HTS is limited, particularly among key populations, this may be because of lack of access, stigma and discrimination (including fear of being marginalized and criminalized), lack of awareness or cost and affordability [6]. To address this gap, social network‐based approaches (SNAs) may be an additional strategy to increase the uptake of HIV testing interventions and improve HIV case‐finding [6].

SNA refers to a process whereby individuals act as “test promoters” (sometimes called “seeds”) who recruit and motivate sexual or injecting partners and/or those in their social networks who may benefit from HIV testing to participate in HTS [7]. Test promoters include those newly diagnosed with HIV, established acquisition or HIV negative but determined to be at higher risk. A social network refers to a group of individuals linked by a common set of relationships or behaviours. These networks can exist in the physical or virtual space and, in the context of HIV, may include sexual, drug injecting or social contacts [7, 8]. One of the benefits of this approach to HTS uptake may be that the request to test comes from a trusted community member. For example, it was possible to effectively distribute HIVST kits to African American and Latino MSM using social network strategies, particularly those who had never tested and were more likely to be living with HIV compared with standard testing strategies [9]. However, there were settings where social network approaches may not yield a higher new positivity rate compared to other testing strategies [10].

In 2019, the WHO made a conditional recommendation based on very low certainty evidence that SNA can be offered as an approach to HIV testing for key populations as part of a comprehensive package of care and prevention [11]. The evidence was evaluated using the GRADE (Grading of Recommendations, Assessment, Development, and Evaluations) framework, which provides certainty in evidence beyond reliance on significant *p*‐values [12]. Certainty of an outcome is assessed using five domains (risk of bias, inconsistency, indirectness, imprecision and publication bias), and presented as high, moderate, low or very low certainty. Evidence showed that SNA may increase HIV diagnoses and identify additional people with HIV, is acceptable, is feasible to implement, is an efficient use of resources when focused on people with high ongoing HIV risk and results in very few minor social harms or adverse events. However, it was noted that these data should be interpreted cautiously due to no randomized trials and the very low certainty of the evidence. Notably, all data were collected and analysed in the context of targeting key populations, specifically, MSM, PWID, people in prisons or other closed settings, sex workers and trans and gender‐diverse people, and did not include all individuals who could potentially benefit from HTS [11].

Following the 2019 WHO guidelines, SNA has been introduced in national policies (e.g. Colombia, New Zealand, Thailand) and reported to be a successful way to reach key populations [13]. Programmes are now seeking ways to apply the lessons learned from this approach to expand HTS further. We conducted a systematic review to inform the 2023 WHO guidelines development process following the GRADE approach [14]. Our aim was to assess the effectiveness, acceptability and cost‐effectiveness of SNA for HIV testing across the general and key populations.

## 2 METHODS

### 2.1 Search strategy

This systemic review was conducted in accordance with the Cochrane Handbook for Systemic Reviews of Interventions [15] and followed the Preferred Reporting Items for Systemic Reviews and Meta‐Analyses (PRISMA) guidelines for reporting [16]. First, a comprehensive search and screening process was performed, followed by data extraction from primary studies. Next, we assessed the risk of bias for included studies. Finally, we synthesized the results and evaluated the certainty of evidence using the GRADE approach, where appropriate. We registered our protocol in the International Prospective Register of systematic reviews (PROSPERO, CRD42022351233).

We searched the following databases without language restriction: Medline, Embase, Global Health, CINAHL, Web of Science, PsycINFO and PubMed. To capture recent data, we limited our search from 1 Jan 2010 to 22 July 2022, which was updated on 5th May 2023. Our search strategy included the concepts of “HIV,” “testing” and “social networks.” Further details of our search strategy are provided in Supplementary 1. References cited by the studies selected from the database search were manually screened to identify any relevant papers missed by the search strategy. Search results from each database were merged, and any duplication of studies was removed electronically. Two reviewers (AC and YL) independently screened titles and abstracts using the Covidence systematic review software (Veritas Health Innovation, Melbourne, Australia). Any discrepancies were resolved by a third reviewer (JO).

We included randomized trials and non‐randomized studies that compared SNA with non‐SNA approaches (i.e. any HTS that did not include SNA or no intervention) or that compared different models of SNA. Non‐SNA approaches were mostly described as venue‐based testing, but some variations exist in studies (further details in Supplementary 5). There were no restrictions on population type (e.g. key populations or general population) or geographical location. The primary outcomes of interest were divided into three main themes: effectiveness, acceptability and cost‐effectiveness. We measured effectiveness as: (1) Proportion of people offered SNA (i.e. test promoters) who accepted participating; (2) Uptake of HTS among partners/social contacts of test promoters; (3) Proportion of first‐time testers among partners/social contacts of test promoters; (4) Percentage of people newly tested positive for HIV; (5) Baseline CD4 count or viral load among people diagnosed with HIV; (6) Proportion linked to services; (7) Identifying people with HIV who are not engaged in care (e.g. not on antiretroviral therapy (ART), or not virally suppressed). We measured acceptability as: (8) Social harms or adverse events among test promoters or partners/social contacts; (9) Acceptability of SNA among test promoters and their partners/social contacts; and (10) Cost‐effectiveness. We measured cost‐effectiveness through any data for resource use, including cost‐effectiveness analysis. We also extracted secondary outcomes to better understand implementation considerations: population type, study design, number of waves of recruitment and whether financial incentives were offered as part of SNA.

### 2.2 Data extraction

Eligible full‐text articles were assessed independently by two reviewers for data extraction using a data extraction tool in an Excel spreadsheet, and a third reviewer checked and reconciled all differences in data extraction. Ten relevant studies underwent preliminary extraction to assess the appropriateness of the data extraction spreadsheet, and when necessary, modified and adapted to capture more relevant information from studies.

### 2.3 Quality assessment

The Cochrane Risk‐of‐Bias tool for Randomised trials (RoB 2) for randomised controlled trials (RCTs) [17] and ROBINS‐I [18] for non‐randomized studies were used to evaluate the risk of bias in studies included in the effectiveness review. For qualitative studies, we used the critical appraisal skills programme (CASP) Qualitative Studies checklist [19].

### 2.4 Data analysis

We described the details of the SNA and non‐SNA approaches from each study (Supplementary 5). For studies that shared similar interventions, control and outcomes, we used Review Manager (RevMan, version 5.4, The Cochrane Collaboration, 2020) to conduct the random‐effects meta‐analysis. We used the Mantel‐Haenszel method for calculating risk ratios as the estimates of the standard errors may be more precise than other methods (e.g. inverse variance methods) when data are relatively sparse [20]. We calculated the pooled proportions and 95% confidence intervals (CI) for outcomes 1–7 as described above. We assessed statistical heterogeneity between studies with the *I* ^2^ statistic. To further explore the variability in SNA approaches, we conducted subgroup meta‐analyses based on the following pre‐defined covariates: population type, study design, number of waves of recruitment and whether financial incentives were offered as part of SNA. Forest plots were created to visualize the results of the meta‐analyses. Where more than one type of SNA was evaluated, we specified this in the forest plots. Funnel plots were created to assess the potential for publication bias. We evaluated the risk of bias, precision, directness and certainty of the evidence to determine an overall certainty of evidence (high, moderate, low, very low) per critical outcome using GRADEPro software (gradepro.org) and formulated Evidence Profile summary tables for each outcome. We report effectiveness using the recommended approach where the level of certainty and clinical or public health importance of the effect estimate determine the interpretation of the effect estimate [21].

For qualitative studies that discussed the acceptability of SNA among test promoters and their partners/social contacts, social harms or adverse events among test promoters or partners/social contacts, we used the GRADE‐CERQual approach to provide a confidence rating of the certainty of the evidence [22]. Following guidance using the GRADE approach for economic evidence [23], we first summarized the data on costs and resources descriptively. We then identified items of resource use that may differ between alternative management strategies and are potentially important to clients and decision‐makers (e.g. recurrent and fixed costs of personnel time, costs of consumables). We presented data on the cost per person tested and cost per person newly diagnosed with HIV, comparing SNA with non‐SNA.

## 3 RESULTS

**Figure 1:**
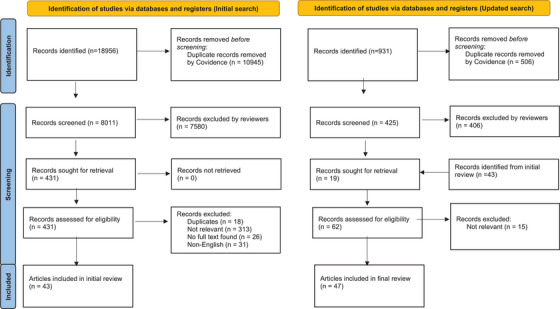
PRISMA flowchart.

Figure 1 summarizes the number of studies identified. We included 47 unique studies with some studies contributing more than one type of data: 15 studies evaluated the effectiveness of SNA versus non‐SNA, 10 studies assessed the effectiveness of different models of SNA, 4 studies reported resource use of SNA versus non‐SNA, 3 studies reported resource use of different models of SNA and 23 studies assessed the acceptability of SNA. The main characteristics of the studies are summarized in Table 1 (with details of each study provided in Table S10). About half of the included studies were non‐randomized studies (45%), from the Americas (53%) and most recruited MSM or transgender women (TGW) (62%). The quality assessment is provided in Supplementary 2, demonstrating almost all non‐randomized studies were marked down in the GRADE evidence profile for risk of bias due to confounding because of the self‐reported outcomes.

**Table 1:** Characteristics of included studies

	Total *n* (%)	SNA versus no SNA *n* (%)	Models of SNA *n* (%)	Qualitative studies *n* (%)
**Type of study**				
RCT	3 (6)	1 (7)	3 (19)	0 (0)
Non‐randomized studies	21 (45)	14 (93)	13 (81)	0 (0)
Qualitative	23 (49)	0 (0)	0 (0)	23 (100)
**Latest year of study recruitment**				
2010–2016	23 (49)	10 (67)	7 (44)	9 (39)
2017–2023	24 (51)	5 (33)	9 (56)	14 (61)
**Study site**				
African Region	13 (28)	0 (0)	4 (25)	9 (39)
Region of Americas	25 (53)	12 (80)	6 (38)	11 (48)
European Region	2 (4)	1 (7)	1 (6)	1 (4)
Western Pacific Region	7 (15)	2 (13)	5 (31)	2 (9)
**Population type**				
MSM/TGW	29 (62)	11 (73)	9 (56)	14 (61)
General population	16 (34)	3 (20)	6 (38)	9 (39)
PWID	1 (2)	1 (7)	1 (6)	0 (0)

Abbreviations: MSM, men who have sex with men; PWID, people who inject drugs; RCT, randomized controlled trial; SNA, social network‐based approaches; TGW, transgender women.

### 3.1 Effectiveness of SNA

#### 3.1.1 Proportion of people offered SNA who accepted participating (Outcome 1)

We did not find relevant studies.

#### 3.1.2 Uptake of HTS among partners/social contacts of test promoters (Outcome 2)

We identified six studies: one RCT [24] and five non‐randomized comparator studies [25, 26, 27, 28, 29]. The RCT from the United States reported an uptake of HIV testing as 43.9% (25/57) in the SNA group, where participants were assigned to a Facebook group with a test promoter who delivered information about HIV testing and prevention compared with 20.0% (11/55) in the control group, where participants were assigned to a Facebook group with a test promoter who delivered information about general health [24]. Three non‐randomized comparator studies among MSM reported an uptake of HIV testing among partners/social contacts of test promoters of 98.2% (10,953/11,157) in the SNA group compared with 27.3% (4054/14,865) in the comparator group, where participants of HIV testing were recruited through a municipal hospital website, at various venues frequented by MSM/TGW, and through online social media [25, 28, 29]. One non‐randomized comparator study among the general population in the United States reported the uptake of HIV testing as 98.6% (477/484) in the SNA group, where participants received HIV testing and recruitment training as part of respondent‐driven sampling, compared with 95.5% (192/201) in the comparator group, where participants were recruited during random visits to specific gathering venues [26]. One non‐randomized comparator study among “high risk” women (the study did not detail inclusion criteria for who should be considered a high‐risk woman) in Mexico reported an uptake of HIV testing as 26.3% (66/251) in the SNA group, where participants were referred to a testing centre by test promoters selected by community leaders, compared with 12.0% (11/92) in the comparator group, where community leaders directly encouraged other women to access free HIV testing [27].

**Figure 2:**
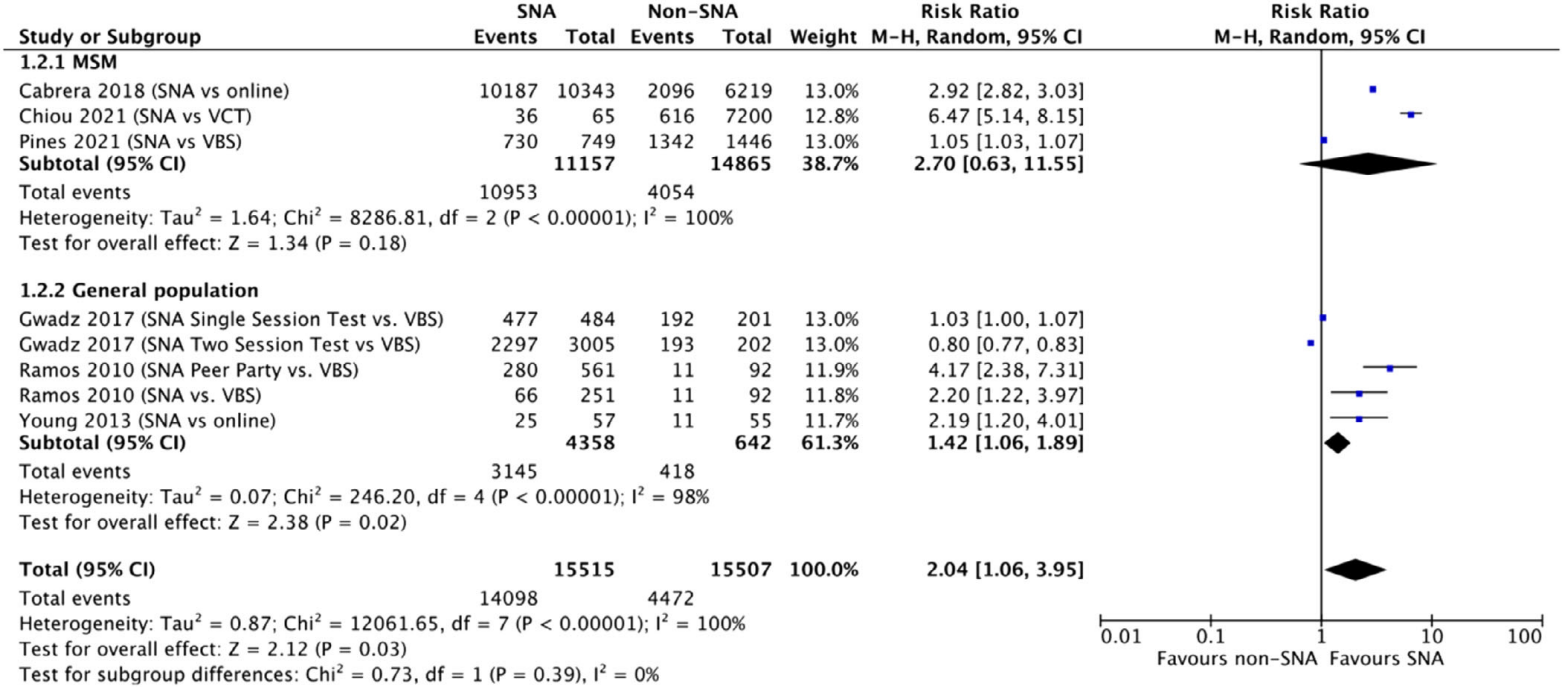
Uptake of HIV testing among partners or social contacts of test promoters. Peer party = gathering organized by test promoters where their social contacts can come together in a safe and comfortable environment to talk about HIV and receive testing; Single session tests = potential test promoters needed to attend one training session before being eligible to be a test promoter; SNA = social network‐based approach; Two session test = potential test promoters were required to attend two training sessions before being eligible to be a test promoter; VCT = voluntary counselling and testing; VBS = venue‐based screening.

A meta‐analysis of these studies determined there was low certainty evidence that SNA may increase uptake when compared to alternative testing options (Pooled RR 2.04, 95% CI: 1.06–3.95, *I* ^2^ = 100%) (Figure 2). Variability of effects was observed by population and service delivery model, with SNA potentially achieving the highest uptake when implemented among MSM and if implemented as part of peer‐led parties among higher‐risk women. A peer‐led party is a gathering organized by test promoters where their social contacts can come together in a safe and comfortable environment to talk about HIV and receive testing. Supplementary 4 and 5 provide further details of the GRADE assessment, pooled risk ratios and a summary of the details of each SNA approach.

#### 3.1.3 Proportion of first‐time testers among partners/social contacts of test promoters (Outcome 3)

We identified eight non‐randomized comparator studies. For MSM, five studies reported the proportion as 28.1% (217/772) in the SNA group compared with 9.0% (289/3209) in the comparator group [9, 25, 30, 31, 32]. For the general population, the two studies reported the proportion as 16.2% (765/4721) in the SNA group compared with 26.4% (3959/14,998) in the comparator group [26, 27]. For PWID, one study reported the proportion as 2.4% (92/5660) in the SNA group, where tested individuals were given coupons to recruit their social contact, compared with 1.4% (56/4640) in the comparator group, where participants were recruited through direct outreach by peers who are living with HIV or former/current PWID [33].

**Figure 3:**
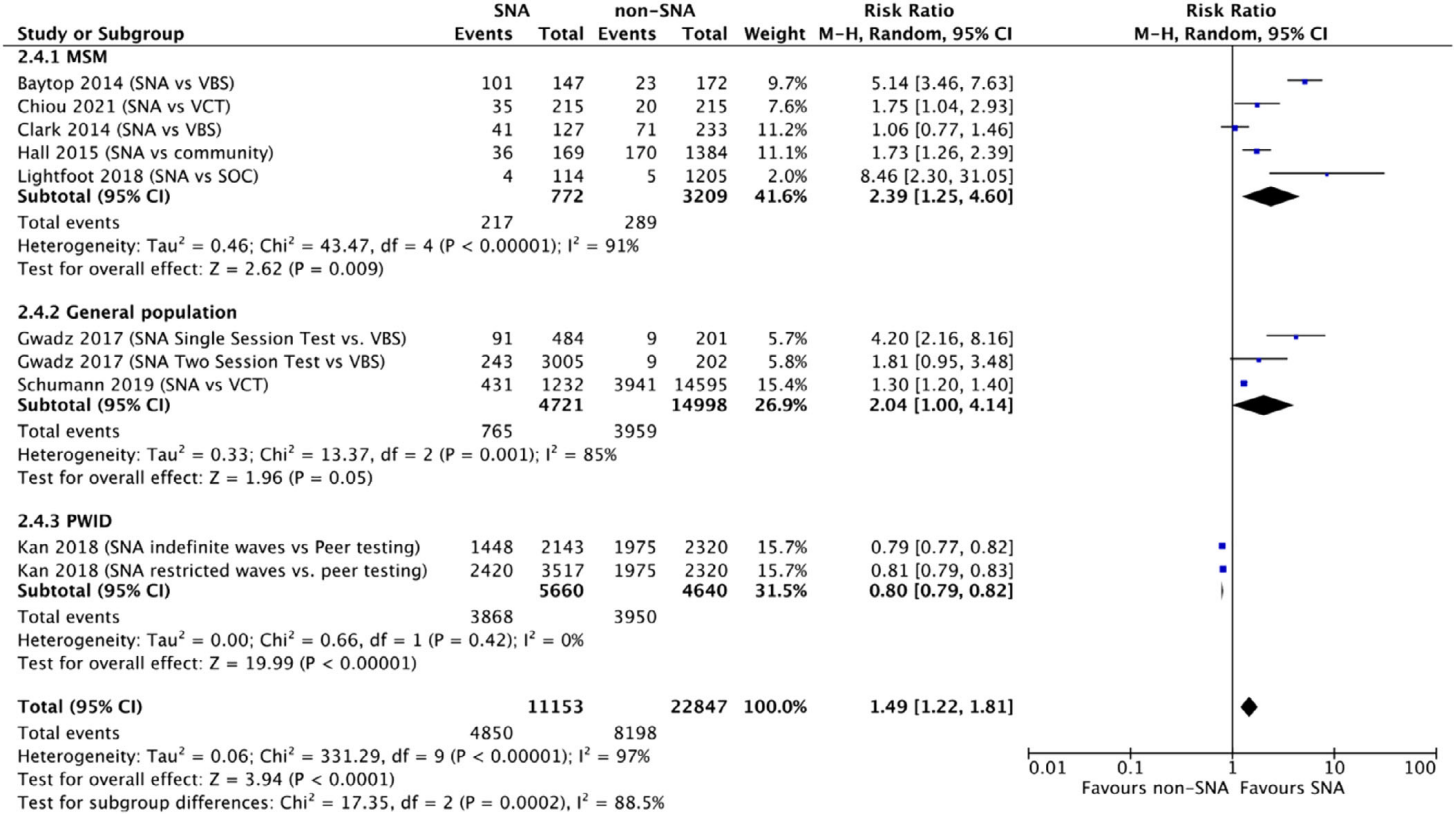
Proportion of first‐time testers among partners or social contacts of test promoters. SNA = social network‐based approach; SNA indefinite waves = number of waves for testing were unrestricted; SNA restricted waves = studies imposed restrictions on the number of waves, for example if two successive individuals were recruited who were HIV negative, no coupons were provided for further recruitment; SOC = standard‐of‐care; VBS = venue‐based testing; VCT = voluntary counselling and testing.

A meta‐analysis of these studies determined there was moderate certainty evidence that SNA probably increased the proportion of first‐time testers when compared to alternative testing options (RR 1.49, 95% CI: 1.22–1.81, *I* ^2^ = 97%) (Figure 3). This large heterogeneity was explained by the population type and the comparator. SNA (compared to venue‐based testing) probably increased the proportion of first‐time testers for MSM (RR 2.39, 95% CI: 1.25–4.60, *I* ^2^ = 91%, Moderate certainty) and might increase the proportion of first‐time testers for the general population (RR 2.04, 95% CI: 1.00–4.14, *I* ^2^ = 85%, Low certainty). However, SNA approaches (compared with peer‐testing) for PWID may decrease the proportion of first‐time testers (RR 0.81, 95% CI: 0.79–0.83, *I* ^2^ = 0%, Low certainty). Here, peer‐testing involved training peers to actively approach the community for HIV testing, whereas SNA uses test promoters to offer testing to their known social contacts.

#### 3.1.4 Proportion of people newly tested positive for HIV among partners/social contacts of test promoters (Outcome 4)

We identified 13 non‐randomized comparator studies. For MSM, 10 studies reported the proportion as 3.5% (803/22,871) in the SNA group compared with 1.3% (915/70,004) in the comparator group [9, 25, 28, 29, 30, 31, 32, 34, 35, 36]. For the general population, two studies reported the proportion as 1.0% (49/4, 721) in the SNA group compared with 0.5% (72/14,998) in the comparator group [10, 26]. For PWID, one study reported the proportion as 2.6% (90/3517) in the SNA group, where tested individuals were given coupons to recruit their social contact, compared with 1.5% (34/2320) in the comparator group where participants were recruited through direct outreach by peers who are living with HIV or former/current PWID [33].

**Figure 4:**
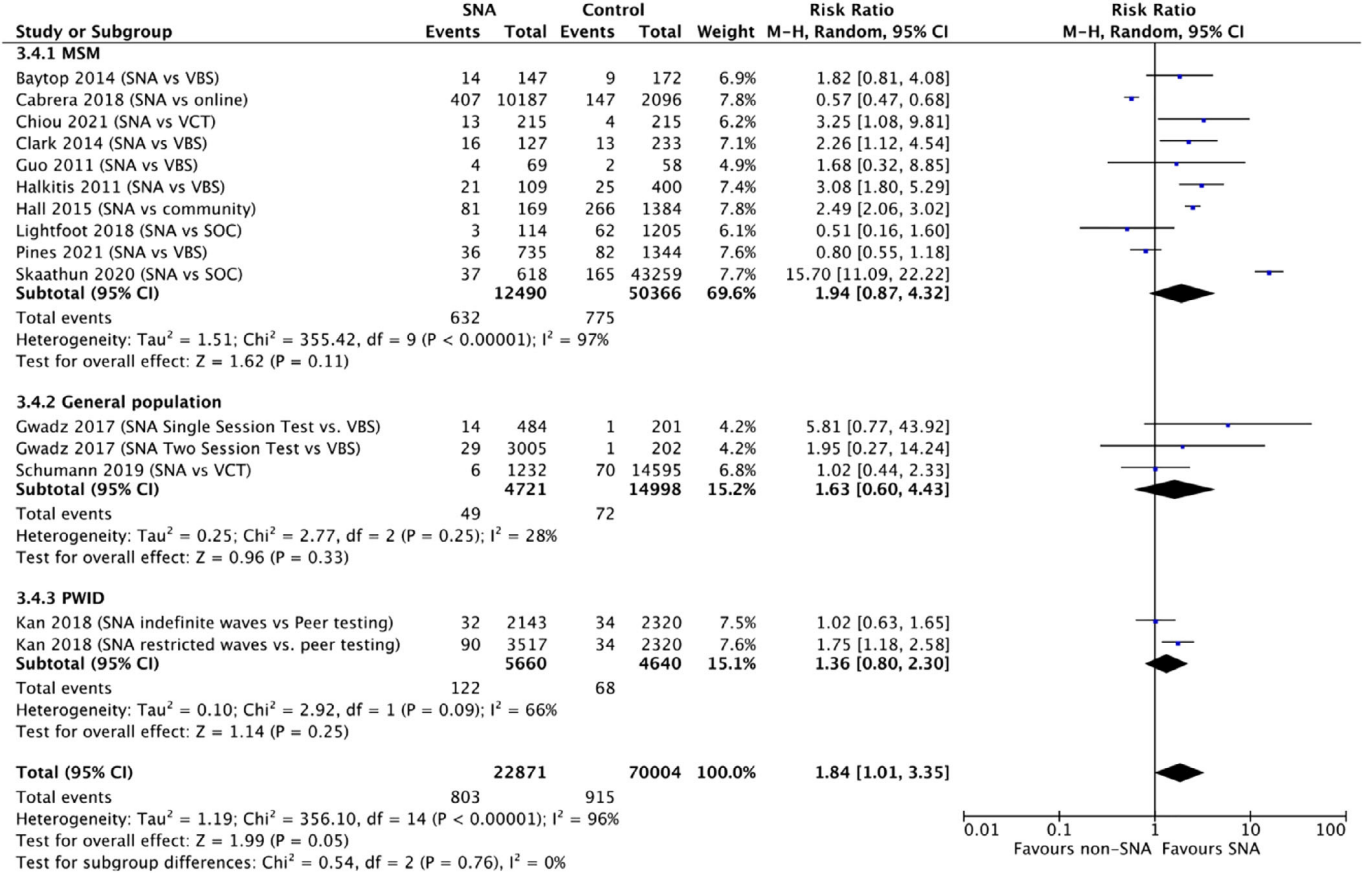
Proportion of people who tested positive among partners or social contacts of test promoters.

A meta‐analysis of these studies determined there was low certainty evidence that SNA may increase the proportion of newly tested positive compared to alternative testing options (RR 1.84, 95% CI: 1.01–3.35, *I* ^2^ = 96%) (Figure 4). High heterogeneity was partly explained by population type and type of SNA.

#### 3.1.5 Baseline CD4 count or viral load among people diagnosed with HIV (Outcome 5)

We did not find any relevant studies.

#### 3.1.6 Proportion of people newly tested positive who were linked to care (Outcome 6)

We identified two non‐randomized comparator studies for MSM [25, 29]. The proportion was reported as 53.3% (224/420) in the SNA group compared with 62.9% (95/151) in the comparator group [25, 29].

There was low certainty evidence that SNA may make little difference in the proportion of people newly tested positive linked to care compared to alternate testing options (RR 1.37, 95% CI: 0.33–5.71, *I* ^2^ = 68%) (Figure S1). Figures S2–S4 are the funnel plots, suggesting a possibility of publication bias for the uptake of HIV testing and the proportion of first‐time testers.

#### 3.1.7 Identifying people with HIV who are not engaged in care (Outcome 7)

We did not find any relevant studies.

### 3.2 Acceptability

There were no reports of any adverse events or harms associated with SNA (Outcome 8) in data from RCTs and non‐randomised studies (NRS) identified in this review. However, we identified some perceptions and concerns about potential harm in nine qualitative studies [37, 38, 39, 40, 41, 42, 43, 44, 45, 46, 47, 48]. Key concerns included fears about the potential for fear of intimate partner violence, relationship dissolution, discrimination and stigma (low confidence). Although many liked the idea of offering SNA with HIVST kits, some had concerns that providing a kit to a partner could be perceived as a sign of mistrust (high confidence) [38, 48].

Based on 23 qualitative studies, we found that SNA is likely to be acceptable (Outcome 9) to a variety of populations (3 for female sex workers [49, 50, 51], 2 for adolescents and young women [41, 45, 48], 1 for youth [52], 12 for MSM/TGW [39, 40, 42, 43, 44, 46, 53, 54, 55, 56, 57, 58, 59, 60] and 3 for the general population [37, 38, 61]). Overall, SNA was perceived as empowering, enabling opportunities for discussion about sexual health and facilitating joint testing. Further details on SNA acceptability by population are provided in Supplementary 7.

### 3.3 Cost‐effectiveness

Cost‐effectiveness evidence (Outcome 10) was based on six studies (five for MSM [28, 34, 62, 63, 64] and one for the general population [65]). All used micro‐costing approaches (i.e. ingredients‐based or a bottom‐up approach) to comprehensively capture costs related to start‐up, fixed and variable costs. Conclusions regarding costs were of moderate certainty due to the limitations of the study design, that is selection bias from non‐random allocation of participants. We did not identify studies with reports on cost per QALY gained/DALY saved. Cost‐effectiveness was based on the cost per person tested and the cost per person newly diagnosed with HIV (Table 2). Further details on resource use are provided in Supplementary 6. An additional study for the general population in Mexico reported that using test promoters to spread health messages through their social networks rather than being disseminated from a single trained community leader using a one‐on‐one approach might reduce the mean staff hours per person testing: SNA (6.18 hours), SNA with testing parties (3.68 hours) and one‐on‐one outreach (22.7 hours) [27].

**Table 2:** Summary of cost‐effectiveness studies that compares SNA with other approaches

Study	Setting	Currency (year)	Cost per person tested	Cost per person diagnosed
Skaathun et al. [34]	USA, MSM	USD (2016)	SNA: $1142 Partner services: $2857 Hospital‐based testing: $48	SNA: $15,683 Partner services: $61,418 Hospital‐based testing: $16,773
Pines et al. [28]	Mexico, MSM/TGW	USD (2017)	SNA: $260 Venue‐based testing: $234	SNA: $5273 Venue‐based testing: $3828
Zulliger et al. [62]	USA, MSM	USD (2014)	SNA: $584 Venue‐based testing: $112	SNA: $16,949 Venue‐based testing: $3823
Zhou et al. [63]	China, MSM	USD (2020)	SNA + Financial incentive (FI): $62 SNA + FI + Peer referral link: $42 SNA + No FI: $96	SNA + Financial incentive (FI): $1555 SNA + Financial incentive + Peer referral link: $1538 SNA + No Financial incentive: $930
Sha et al. [64]	China, MSM	USD (2020)	SNA + HIVST: $120 SNA + testing referral cards: $9408	SNA + HIVST: $2348 SNA + testing referral cards: NA
Shahmanesh et al. [65]	South Africa, General population	USD (2019)	SNA: $36 (per kit distributed) Peer‐navigator distribution HIVST: $56 Standard of care: $64	

Abbreviations: HIVST, HIV self‐testing; MSM, men who have sex with men; NA, not applicable; SNA, social‐network based approach; TGW, transgender women; USA, United States of America; USD, United States Dollars.

### 3.4 Comparing different models of SNA implementation

For our secondary outcomes, 10 studies (including two RCTs) provided data that compared the effectiveness of different models of SNA implementation. A description of how different models of SNA were implemented is provided in Supplementary 5. These SNA models differed in whether HIVST kits were distributed (compared to information about where to get tested), types of promoters (e.g. recruited from community vs. clinics), rules around number of eligible waves (e.g. a study may stop further recruitment of social network members when two successive individuals were recruited who were HIV negative), use of financial incentives or need for training sessions for test promoters. Figures S5−S8 present the forest plots to summarize the risk ratios for each effectiveness outcome, including examples of where these components were not found to be effective. While we did not identify an ideal SNA model, Table 3 provides a summary of components of SNA that could influence service delivery.

**Table 3:** Summary of studies utilizing different components of SNA

Key variables	Examples of studies	Interpretation
HIV self‐testing	**Increased uptake of SNA**: RR 2.58, 95% CI:1.72–3.88 [64] **Linkage to care:** RR 1.89, 95% CI: 0.26–13.50 [66]	SNA with HIVST may increase the uptake of SNA compared to SNA with referral cards and achieve comparable linkage.
Type of test promoters	**Increased uptake of SNA: ** RR 3.04, 95% CI: 2.95–3.13 [67] Community‐based HIV testing sites versus primary care clinics **Increased first‐time testers:** RR 13.55, 95% CI: 1.76–104.56 [67] Favouring test promoters from primary care clinics **Increased proportion that tested positive:** RR 10.84, 95% CI: 6.93–16.95 [67] Favouring test promoters from primary care clinic test promoters (South Africa), but the type of test promoter did not have a statistically significant effect in Malawi for the same outcomes [68].	The type of test promoters used in SNA may impact the uptake of SNA, the proportion of first‐time testers and the proportion that tested positive. However, this could vary depending on the country setting.
Number of waves of recruitment	**Increased proportion that tested positive**: RR 1.29, 95% CI: 1.26–1.32 [26] **Increased first‐time testers: ** RR 2.33, 95% CI: 1.86–2.90 [26] RR 13.55, 95% CI: 1.76–104.56 [67] **Variable HIV positivity** Could improve when stopped after two consecutive HIV‐negative individuals [33]	SNA with multiple waves increased uptake and first‐time testers. Variability or no effect across studies with single waves. Variable or no impact on positivity by wave. Fewer waves could be less costly overall, but more waves could be important to lower the cost per diagnosis if well‐targeted.
Offering financial incentives	**Increased uptake of SNA: ** RR 3.04, 95% CI: 2.95–3.13 [67] RR 1.90, 95% CI: 1.52–2.37 [27] RR 1.42, 95% CI: 1.16–1.75 [66] **Increased first‐time testers: ** RR 13.55, 95% CI: 1.76–104.56 [67] **Increased proportion that tested positive: ** RR 10.84, 95% CI: 6.93–16.94 [67]	SNA without financial incentives still had high uptake, increased first‐time testers and increased proportion that tested positive. Uptake, first‐time testers and positivity among studies with financial incentives varied or had no difference.
Training sessions	**Increased uptake of SNA**: RR 1.29, 95% CI: 1.26–1.29 [26]	SNA with a single session had increased uptake compared to two sessions. Fewer sessions are likely less costly. Fewer sessions are likely more feasible and acceptable.

Abbreviations: RR, relative risk; SNA, social network‐based approach; 95% CI, 95% confidence intervals.

### 3.5 Studies with separate TGW data

Although TGW was a population group of interest, we only identified a few studies that reported TGW data independent from the MSM cohort. Of five quantitative studies that included TGW data, only one study [28] reported it separately from MSM data. The authors reported no significant difference between MSM and TGW for uptake of HIV testing among partners/social contacts of test promoters (SNA: MSM 718/737, TGW 12/12, *p* = 0.54; venue‐based testing: MSM 1256/1349, TGW 86/97, *p* = 0.14) but potentially higher proportion of people newly tested positive for HIV for TGW (SNA: MSM 34/718, TGW 2/12, *p* = 0.06; venue‐based testing: MSM 71/1256, TGW 11/86, *p* = 0.01). For the qualitative outcomes, of five studies that recruited TGW, three studies presented TGW responses [42, 44, 46, 59].

## 4 DISCUSSION

Our review collated evidence for SNA to HIV testing covering the general population who may benefit from HIV testing (not just key populations, as in the previous analysis [11]) and summarized new evidence for the effectiveness, acceptability and cost‐effectiveness of different models of SNA. Based on the included studies, our analyses suggest SNA may be an effective, acceptable and cost‐effective HIV testing approach. While we did not identify an ideal model of SNA that could be immediately scaled up, for each setting and population targeted, we recommend various implementation considerations as our meta‐analysis showed the effectiveness might differ due to factors which include the testing modality (i.e. use of HIVST), type of test promoters, number of waves of recruitment, offer of financial incentives or need for training sessions.

We observed several key considerations for the successful implementation of SNA, although it was difficult to separate the specific impact of separate SNA elements. While these comparative studies demonstrated superiority using certain SNA elements, these can be context‐specific, and their effectiveness was not always consistent across populations and settings. First, the testing modality with which SNA is delivered could increase uptake. For example, studies with HIVST engaged more SNA uptake than testing card referrals [64], consistent with prior evidence suggesting HIVST can reach those who may not otherwise test due to restrictive environments or other barriers [69]. Where opportunities arise, combining dual self‐testing for syphilis and HIV might increase participants’ willingness to access and distribute self‐tests, consistent with prior studies suggesting testers prefer to integrate HIV testing with other sexually transmitted infection (STI) testing [70]. While there are limited data to evaluate the linkage to care post HIVST using an SNA approach, a recent systematic review of 15 studies identified no significant difference between linkage to care post HIVST and facility‐based testing [71].

Second, community‐led engagement in selecting test promoters is important for the successful implementation of SNA. For example, Kitenge et al. [67]. found that the acceptability of HIVST distribution was thrice as high in community‐based testing sites compared to primary health clinics. This is also consistent with a recent network analysis in Kenya [72] which highlighted communities’ important role in HIV prevention, reporting a positive association between community‐based organization membership and increased access to health services. However, we found mixed evidence on how to find test promoters who are likely to participate and offer HIV testing to relevant social contacts. For example, Rosenberg et al. [68]. reported no significant difference in SNA uptake between test promoters recruited from the community or STI clinic. However, contacts of clinic test promoters living with HIV were more likely to also have HIV than contacts of community test promoters who did not have HIV (31% vs. 11%). Previous studies indicate those who engage in HIV higher risk behaviours have more ties with others who engage in similar norms, attitudes and HIV risk activities [73, 74, 75], which might explain this finding. Therefore, depending on local contexts, involving the community in deciding which test promoters to involve in SNA is important.

Third, depending on resource constraints, implementers of SNA may consider the number of waves of recruitment. We did not find a consistent pattern as different studies used different approaches with mixed results. For example, in a study from the United States among the general population that allowed multiple waves, social contacts were encouraged to recruit a further three to five social contacts and reported that this significantly increased the proportion of first‐time testers and those who tested positive [26]. However, a study from South Africa among the general population that used a single wave approach found this significantly increased the proportion of first‐time testers, but the study did not explore if there were additional benefits of allowing multiple waves [67]. Utilizing multiple waves may increase the chance of recruiting hidden populations at risk of HIV who are usually marginalized and/or whose behaviour is criminalized. Longer recruitment chains allow for deeper penetration into the target population and increase the chance of the sample becoming independent of the test promoters, thereby overcoming differential recruitment patterns [76]. Notably, there were also examples of more targeted recruitment. Among PWID, Kan et al. [33]. reported that stopping recruitment when there were two HIV‐negative recruits may increase the yield of active case‐finding relative to indefinite waves. However, this approach may need greater resources for recruitment of test promoters, since recruitment chains are ended more quickly.

Fourth, while qualitative data indicated financial incentives might facilitate SNA uptake for youths (moderate confidence), SNA without financial incentives still achieved high uptake of HTS, increased first‐time testers and increased proportion that tested positive. This has important implications in settings with financial constraints where SNA can still be effective without providing financial incentives. There may be implementation challenges of financial incentives, including equity, sustainability and universal health coverage initiatives, and further research on non‐cash incentives may overcome these challenges.

Finally, Ramos et al. [27]. implemented “testing parties” for women in Mexico, held at the test promoters’ homes to provide a safe, comfortable environment for the women to receive HIV education and testing. They found these parties more successful in increasing HTS than one‐on‐one SNA, as changing social norms to normalize HTS may improve testing behaviours. Similarly, a pilot study recruiting African American women in the United States demonstrated house parties as an intervention venue can encourage women to support each other and provide opportunities to discuss issues around sexual control, condom negotiation and increase overall HIV knowledge [77]. However, this study did not offer rapid testing with concerns around women finding out a possible positive test result among their friends, which could challenge confidentiality, and perhaps a follow‐up to disclose test results after parties would be optimal.

This review should be read considering some limitations. According to our GRADE assessment, the evidence overall had a low certainty; however, the benefits are likely to outweigh the risks of SNA. Caution is warranted when interpreting resource use as different denominators (e.g. size of programmes) affected the drivers of cost (i.e. personnel time). No studies were identified that included baseline CD4 count or viral load among people diagnosed with HIV, re‐engagement in care, and the geographic location of the data was not comprehensive, so further research is warranted to improve the generalizability of findings. Lastly, most studies arose from North America and involved MSM/TGW, which may decrease the generalizability across different cultures, financial and social settings. More studies are needed from general populations and low‐ and middle‐income countries (LMICs). This underscores the importance of context‐specific evaluations before SNA is scaled up. As per our review of qualitative studies (Supplementary 7), each context revealed specific socio‐cultural factors that may influence the potential effectiveness and acceptability of SNA. This underscores the importance to carefully evaluate the effectiveness and cost‐effectiveness of SNA within a specific setting before further resources are invested to scale up the approach.

## 5 CONCLUSIONS

In conclusion, we found evidence of the potential for social network‐based approaches to significantly enhance HIV testing uptake, increase the proportion of first‐time testers and those testing positive for HIV. While the heterogeneity among studies highlights the need for context‐specific adaptations, the overall positive impact of SNA on HIV testing outcomes could support its integration into existing HTS. As we strive to meet global HIV targets, incorporating SNA can bridge the gaps in current HIV testing efforts, particularly in reaching underserved populations.

## COMPETING INTERESTS

CCJ, RB, MB‐D, MSJ and VM are World Health Organization staff members. JJO has received research funding from Gilead to their institution outside of the submitted work. EPFC has received research funding to their institution from Merck and Seqirus outside of the submitted work. MB‐D and MSJ have received research funding to their institution from BMGF and received funding to WHO from USAID outside of the submitted work.

## AUTHORS’ CONTRIBUTIONS

CCJ, RB and JJO conceived the idea. AC, YML and JJO did the screening, data extraction and wrote the first draft of the manuscript. JJO conducted the statistical analysis. EPFC accessed the analysed data and verified the analyses. NLS and CCJ reviewed and supported the GRADE analysis. Collaborators from WHO were involved in study design, interpretation of results and manuscript development; no other funders had a role in design, analysis or interpretation. All collaborators had full access to all the data in the study. JJO had final responsibility for the decision to submit for publication, with concurrence from all authors.

## DISCLAIMER

The authors alone are responsible for the views expressed in this publication, and they do not necessarily represent the views, decisions or policies of the institutions with which they are affiliated.

## Supporting information


**Table S1**. Search terms—EMBASE
**Table S2**. Search terms—Medline
**Table S3**. Search terms—Global Health Database
**Table S4**. Search terms—PsycINFO
**Table S5**. Search terms—PubMed
**Table S6**. Search terms—EBSCO CINAHL
**Table S7**. Search terms—Web of Science
**Table S8**. Risk Of Bias in Non‐randomised Studies—of Interventions
**Table S9**. Version 2 of the Cochrane risk‐of‐bias tool for randomised trials (RoB 2)
**Table S10**. Description of types of SNA models
**Table S11**. Costs for SNA versus non‐SNA
**Table S12**. Costs for types of SNA
**Table S13**. Cost‐effectiveness for types of SNA
**Table S14**. Summary of qualitative findings
**Figure S1**. Proportion of people who tested positive among partners or social contacts of test promoters who linked to care
**Figure S2**. Funnel plot of uptake of HIV testing among partners or social contacts of test promoters
**Figure S3**. Funnel plot for the proportion of first‐time testers among partners or social contacts of test promoters
**Figure S4**. Funnel plot of the proportion of people who tested positive among partners or social contacts of test promoters
**Figure S5**. Uptake of SNA for test promoters
**Figure S6**. Uptake of HIV testing among partners or social contacts of test promoters
**Figure S7**. Proportion of first‐time testers among partners or social contacts of test promoters
**Figure S8**. Proportion of people tested positive among partners or social contacts of test promoters
Supplementary 4 GRADE evidence profile
Supplementary 7 Qualitative data

## Data Availability

Data will be made available upon request made to the corresponding author.
