# Supplementary material for: Social network‐based approaches to HIV testing: a systematic review and meta‐analysis
Source: J Int AIDS Soc. 2024 Sep 9;27(9):e26353. doi: 10.1002/jia2.26353 (PMC11386243; doi:10.1002/jia2.26353)
Supplement: Supplementary file 1 — Table S1. Search terms—EMBASE Table S2. Search terms—Medline Table S3. Search terms—Global Health Database Table S4. Search terms—PsycINFO Table S5. Search terms—PubMed Table S6. Search terms—EBSCO CINAHL Table S7. Search terms—Web of Science Table S8. Risk Of Bias in Non‐randomised Studies—of Interventions Table S9. Version 2 of the Cochrane risk‐of‐bias tool for randomised trials (RoB 2) Table S10. Description of types of SNA models Table S11. Costs for SNA versus non‐SNA Table S12. Costs for types of SNA Table S13. Cost‐effectiveness for types of SNA Table S14. Summary of qualitative findings Figure S1. Proportion of people who tested positive among partners or social contacts of test promoters who linked to care Figure S2. Funnel plot of uptake of HIV testing among partners or social contacts of test promoters Figure S3. Funnel plot for the proportion of first‐time testers among partners or social contacts of test promoters Figure S4. Funnel plot of the proportion of people who tested positive among partners or social contacts of test promoters Figure S5. Uptake of SNA for test promoters Figure S6. Uptake of HIV testing among partners or social contacts of test promoters Figure S7. Proportion of first‐time testers among partners or social contacts of test promoters Figure S8. Proportion of people tested positive among partners or social contacts of test promoters Supplementary 4 GRADE evidence profile Supplementary 7 Qualitative data [file JIA2-27-e26353-s001.docx]

**Table of contents**

Supplementary 1. Search strategy 5

Table S1. Search terms – EMBASE 5

Table S2. Search terms – Medline 7

Table S3. Search terms – Global Health Database 9

Table S4. Search terms – PsycINFO 10

Table S5. Search terms – PubMed 11

Table S6. Search terms – EBSCO CINAHL 12

Table S7. Search terms – Web of Science 13

Supplementary 2. Quality Assessment 14

Table S8. Risk Of Bias in Non-randomised Studies - of Interventions 14

Table S9. Version 2 of the Cochrane risk-of-bias tool for randomised trials (RoB 2) 15

Supplementary 3. Forest plots 16

Supplementary Figure 1 Proportion of people who tested positive among partners or social contacts of test promoters who linked to care 16

Supplementary Figure 2 Funnel Plot of uptake of HIV testing among partners or social contacts of test promoters 17

Supplementary Figure 3 Funnel plot for the proportion of first-time testers among partners or social contacts of test promoters 18

Supplementary Figure 4 Funnel plot of the proportion of people who tested positive among partners or social contacts of test promoters 19

Supplementary Figure 5 Uptake of SNA for test promoters 20

Supplementary Figure 6 Uptake of HIV testing among partners or social contacts of test promoters 21

Supplementary Figure 7 Proportion of first-time testers among partners or social contacts of test promoters 22

Supplementary Figure 8 Proportion of people tested positive among partners or social contacts of test promoters 23

Supplementary 4. GRADE evidence profile 24

Supplementary 5. 32

Table S10 Description of types of SNA models 32

Supplementary 6. Resource Use 39

Table S11 Costs for SNA vs. non-SNA 39

Table S12 Costs for types of SNA 39

Table S13 Cost-effectiveness for types of SNA 40

Supplementary 7. Qualitative data 41

Table S14 Summary of Qualitative Findings 42

# Supplementary 1. Search strategy

### Table S1. Search terms – EMBASE

| Search Number | Search strategy |
| --- | --- |
| 1. | exp Human immunodeficiency virus infection/ |
| 2. | exp Human immunodeficiency virus/ |
| 3. | hiv.ti,ab. |
| 4. | hiv-1.ti,ab. |
| 5. | hiv-2.ti,ab. |
| 6. | hiv1.ti,ab. |
| 7. | hiv2.ti,ab. |
| 8. | human immunodeficiency virus.ti,ab. |
| 9. | human immunedeficiency virus.ti,ab. |
| 10. | human immuno-deficiency virus.ti,ab. |
| 11. | human immune-deficiency virus.ti,ab. |
| 12. | human.ti,ab. |
| 13. | immun*.ti,ab. |
| 14. | deficiency virus.ti,ab. |
| 15. | 12 and 13 and 14 |
| 16. | acquired immunedeficiency syndrome.ti,ab. |
| 17. | acquired immuno-deficiency syndrome.ti,ab. |
| 18. | acquired immune-deficiency syndrome.ti,ab. |
| 19. | acquired.ti,ab. |
| 20. | immun*.ti,ab. |
| 21. | deficiency syndrome.ti,ab. |
| 22. | 19 and 20 and 21 |
| 23. | 1 or 2 or 3 or 4 or 5 or 6 or 7 or 8 or 9 or 10 or 11 or 15 or 16 or 17 or 18 or 22 |
| 24. | diagnosis/ |
| 25. | test.ti,ab,de. |
| 26. | testing.ti,ab,de. |
| 27. | tests.ti,ab,de. |
| 28. | tested.ti,ab,de. |
| 29. | screen.ti,ab,de. |
| 30. | screened.ti,ab,de. |
| 31. | screens.ti,ab,de. |
| 32. | screening.ti,ab,de. |
| 33. | exp mass screening/ |
| 34. | exp diagnostic test/ |
| 35. | 24 or 25 or 26 or 27 or 28 or 29 or 30 or 31 or 32 or 33 or 34 |
| 36. | exp contact examination/ |
| 37. | sexual partners.ti,ab. |
| 38. | sexual partner.ti,ab. |
| 39. | partner services.ti,ab. |
| 40. | partner notifcation.ti,ab. |
| 41. | exp social network/ |
| 42. | social network.ti,ab. |
| 43. | social networks.ti,ab. |
| 44. | social networking.ti,ab. |
| 45. | contact tracing.ti,ab. |
| 46. | online network.ti,ab. |
| 47. | online networks.ti,ab. |
| 48. | online networking.ti,ab. |
| 49. | exp sociometric status/ |
| 50. | 36 or 37 or 38 or 39 or 40 or 41 or 42 or 43 or 44 or 45 or 46 or 47 or 48 or 49 |
| 51. | 23 and 35 and 50 |
| 52. | limit 51 to (embase and yr="2010 -Current") |

### Table S2. Search terms – Medline

| Search Number | Search strategy |
| --- | --- |
| 1. | human immunodeficiency virus infection.mp. [mp=title, abstract, original title, name of substance word, subject heading word, floating sub-heading word, keyword heading word, organism supplementary concept word, protocol supplementary concept word, rare disease supplementary concept word, unique identifier, synonyms] |
| 2. | human immunodeficiency virus.mp. [mp=title, abstract, original title, name of substance word, subject heading word, floating sub-heading word, keyword heading word, organism supplementary concept word, protocol supplementary concept word, rare disease supplementary concept word, unique identifier, synonyms] |
| 3. | hiv.ti,ab. |
| 4. | hiv-1.ti,ab. |
| 5. | hiv-2.ti,ab. |
| 6. | hiv1.ti,ab. |
| 7. | hiv2.ti,ab. |
| 8. | human immunodeficiency virus.ti,ab. |
| 9. | human immunedeficiency virus.ti,ab. |
| 10. | human immuno-deficiency virus.ti,ab. |
| 11. | human immune-deficiency virus.ti,ab. |
| 12. | human.ti,ab. |
| 13. | immun*.ti,ab. |
| 14. | deficiency virus.ti,ab. |
| 15. | 12 and 13 and 14 |
| 16. | acquired immunodeficiency syndrome.ti,ab. |
| 17. | acquired immunedeficiency syndrome.ti,ab. |
| 18. | acquired immuno-deficiency syndrome.ti,ab. |
| 19. | acquired immune-deficiency syndrome.ti,ab. |
| 20. | acquired.ti,ab. |
| 21. | immun*.ti,ab. |
| 22. | deficiency syndrome.ti,ab. |
| 23. | 20 and 21 and 22 |
| 24. | diagnosis.mp. [mp=title, abstract, original title, name of substance word, subject heading word, floating sub-heading word, keyword heading word, organism supplementary concept word, protocol supplementary concept word, rare disease supplementary concept word, unique identifier, synonyms] |
| 25. | test.ti,ab. |
| 26. | testing.ti,ab. |
| 27. | tests.ti,ab. |
| 28. | tested.ti,ab. |
| 29. | screen.ti,ab,de. |
| 30. | screened.ti,ab,de. |
| 31. | screening.ti,ab,de. |
| 32. | mass screening.mp. [mp=title, abstract, original title, name of substance word, subject heading word, floating sub-heading word, keyword heading word, organism supplementary concept word, protocol supplementary concept word, rare disease supplementary concept word, unique identifier, synonyms] |
| 33. | diagnostic test.mp. [mp=title, abstract, original title, name of substance word, subject heading word, floating sub-heading word, keyword heading word, organism supplementary concept word, protocol supplementary concept word, rare disease supplementary concept word, unique identifier, synonyms] |
| 34. | 24 or 25 or 26 or 27 or 28 or 29 or 30 or 31 or 32 or 33 |
| 35. | contact examination.mp. [mp=title, abstract, original title, name of substance word, subject heading word, floating sub-heading word, keyword heading word, organism supplementary concept word, protocol supplementary concept word, rare disease supplementary concept word, unique identifier, synonyms] |
| 36. | sexual partners.ti,ab. |
| 37. | sexual partner.ti,ab. |
| 38. | partner services.ti,ab. |
| 39. | partner notification.ti,ab. |
| 40. | partner notifications.ti,ab. |
| 41. | social network.mp. [mp=title, abstract, original title, name of substance word, subject heading word, floating sub-heading word, keyword heading word, organism supplementary concept word, protocol supplementary concept word, rare disease supplementary concept word, unique identifier, synonyms] |
| 42. | social netowrk.ti,ab. |
| 43. | social networks.ti,ab. |
| 44. | social networking.ti,ab. |
| 45. | contact tracing.ti,ab. |
| 46. | online network.ti,ab. |
| 47. | online networks.ti,ab. |
| 48. | online networking.ti,ab. |
| 49. | sociometric status.ti,ab. |
| 50. | 35 or 36 or 37 or 38 or 39 or 40 or 41 or 42 or 43 or 44 or 45 or 46 or 47 or 48 or 49 |
| 51. | 1 or 2 or 3 or 4 or 5 or 6 or 7 or 8 or 9 or 10 or 11 or 15 or 16 or 17 or 18 or 19 or 23 |
| 52. | 34 and 50 and 51 |
| 53. | limit 52 to yr="2010 -Current" |

### Table S3. Search terms – Global Health Database

| Search Number | Search strategy |
| --- | --- |
| 1. | ("human immunodeficiency virus infection" or "human immunodeficiency virus" or "hiv" or "hiv-1" or "hiv-2b" or "hiv1" or "hiv2" or "human immunedeficiency virus" or "human immuno-deficiency virus " or "acquired immunodeficiency syndrome" or "acquired immunedeficiency syndrome" or "acquired immuno-deficiency syndrome" or "acquired immune-deficiency syndrome").mp. [mp=abstract, title, original title, heading words, cabicodes words] |
| 2. | ("diagnosis" or "test" or "testing" or "tests" or "tested" or "screen" or "screened" or "screens" or "screening" or "mass screening" or "diagnostic test").mp. [mp=abstract, title, original title, heading words, cabicodes words] |
| 3. | ("contact examination" or "sexual partners" or "sexual partner" or "partner services" or "partner notification" or "partner notifications" or " social network" or "social network" or "social networks" or "social networking" or "contact tracing" or "online network" or "online networks" or "online networking" or "sociometric status").mp. [mp=abstract, title, original title, heading words, cabicodes words] |
| 4. | 1 and 2 and 3 |
| 5. | limit 4 to yr="2010 -Current" |

### Table S4. Search terms – PsycINFO

| Search Number | Search strategy |
| --- | --- |
| 1. | (human immunodeficiency virus infection or human immunodeficiency virus or hiv or hiv-1 or hiv-2b or hiv1 or hiv2 or human immunedeficiency virus or human immuno-deficiency virus or acquired immunodeficiency syndrome or acquired immunedeficiency syndrome or acquired immuno-deficiency syndrome or acquired immune-deficiency syndrome).mp. [mp=title, abstract, heading word, table of contents, key concepts, original title, tests & measures, mesh word] |
| 2. | (diagnosis or test or testing or tests or tested or screen or screened or screens or screening or mass screening or diagnostic test).mp. [mp=title, abstract, heading word, table of contents, key concepts, original title, tests & measures, mesh word] |
| 3. | (contact examination or sexual partners or sexual partner or partner services or partner notification or partner notifications or social network or social network or social networks or social networking or contact tracing or online network or online networks or online networking or sociometric status).mp. [mp=title, abstract, heading word, table of contents, key concepts, original title, tests & measures, mesh word] |
| 4. | 1 and 2 and 3 |
| 5. | limit 4 to yr="2010 -Current" |

### Table S5. Search terms – PubMed

| (HIV Infections[MeSH] OR HIV[MeSH] OR hiv[tiab] OR hiv-1*[tiab] OR hiv-2*[tiab] OR hiv1[tiab] OR hiv2[tiab] OR human immunodeficiency virus[tiab] OR human immunedeficiency virus[tiab] OR human immuno-deficiency virus[tiab] OR human immune-deficiency virus[tiab] OR ((human immun*[tiab]) AND (deficiency virus[tiab])) OR acquired immunodeficiency syndrome[tiab] OR acquired immunedeficiency syndrome[tiab] OR acquired immuno-deficiency syndrome[tiab] OR acquired immune-deficiency syndrome[tiab] OR ((acquired immun*[tiab]) AND (deficiency syndrome[tiab])))  AND  (diagnosis[sh] OR diagnosis[tw] OR test[tw] OR testing[tw] OR tests[tw] OR tested[tiab] OR screen[tw] OR screened[tw] OR screens[tw] OR screening[tw] OR mass screening[mesh] OR routine diagnostic tests[Mesh])  AND  ("Contact Tracing"[Mesh] OR "Sexual Partners"[Mesh] OR "partner services"[tiab] OR "partner notification"[tiab] OR “partner notifications”[tiab] OR "social network"[tiab] OR "social networks"[tiab] OR “social networking”[tiab] OR “contact tracing”[tiab] OR “sexual network” OR “online network” OR “online networks”[tiab] OR “online networking” OR "Sociometric Techniques"[Mesh] OR "Social Networking"[Mesh]) |
| --- |

### Table S6. Search terms – EBSCO CINAHL

| Search Number | Search Strategy |
| --- | --- |
| 1 | TI human immunodeficiency virus infection OR human immunodeficiency virus OR hiv OR hiv-1 OR hiv-2b OR hiv1 OR hiv2 OR human immunedeficiency virus OR human immuno-deficiency virus OR acquired immunodeficiency syndrome OR acquired immunedeficiency syndrome OR acquired immuno-deficiency syndrome OR acquired immune-deficiency syndrome |
| 2 | TI diagnosis OR test OR testing OR tests OR tested OR screen OR screened OR screens OR screening OR mass screening OR diagnostic test |
| 3 | TI contact examination OR sexual partners OR sexual partner OR partner services OR partner notification OR partner notifications OR social network OR social network OR social networks OR social networking OR contact tracing OR online network OR online networks OR online networking OR sociometric status |
| 4  5  6  7  8 | 1 AND 2 AND 3  AB human immunodeficiency virus infection OR human immunodeficiency virus OR hiv OR hiv-1 OR hiv-2b OR hiv1 OR hiv2 OR human immunedeficiency virus OR human immuno-deficiency virus OR acquired immunodeficiency syndrome OR acquired immunedeficiency syndrome OR acquired immuno-deficiency syndrome OR acquired immune-deficiency syndrome  AB diagnosis OR test OR testing OR tests OR tested OR screen OR screened OR screens OR screening OR mass screening OR diagnostic test  AB contact examination OR sexual partners OR sexual partner OR partner services OR partner notification OR partner notifications OR social network OR social network OR social networks OR social networking OR contact tracing OR online network OR online networks OR online networking OR sociometric status  5 AND 6 AND 7 |
| 9  10 | 4 OR 8  Limit from 2010 to current |

### Table S7. Search terms – Web of Science

| Search Number | Search Strategy |
| --- | --- |
| 1 | TI=(human immunodeficiency virus infection OR human immunodeficiency virus OR hiv OR hiv-1 OR hiv-2b OR hiv1 OR hiv2 OR human immunedeficiency virus OR human immuno-deficiency virus OR acquired immunodeficiency syndrome OR acquired immunedeficiency syndrome OR acquired immuno-deficiency syndrome OR acquired immune-deficiency syndrome) |
| 2 | TI=(diagnosis OR test OR testing OR tests OR tested OR screen OR screened OR screens OR screening OR mass screening OR diagnostic test) |
| 3 | TI=(contact examination OR sexual partners OR sexual partner OR partner services OR partner notification OR partner notifications OR social network OR social network OR social networks OR social networking OR contact tracing OR online network OR online networks OR online networking OR sociometric status) |
| 4  5  6  7  8  9  10 | #3 AND #2 AND #1  AB=(human immunodeficiency virus infection OR human immunodeficiency virus OR hiv OR hiv-1 OR hiv-2b OR hiv1 OR hiv2 OR human immunedeficiency virus OR human immuno-deficiency virus OR acquired immunodeficiency syndrome OR acquired immunedeficiency syndrome OR acquired immuno-deficiency syndrome OR acquired immune-deficiency syndrome)  AB=(diagnosis OR test OR testing OR tests OR tested OR screen OR screened OR screens OR screening OR mass screening OR diagnostic test)  AB=(contact examination OR sexual partners OR sexual partner OR partner services OR partner notification OR partner notifications OR social network OR social network OR social networks OR social networking OR contact tracing OR online network OR online networks OR online networking OR sociometric status)  #7 AND #6 AND #5  #8 OR #4  Limit from 2010 to current |

# Supplementary 2. Quality Assessment

### Table S8. Risk Of Bias in Non-randomised Studies - of Interventions


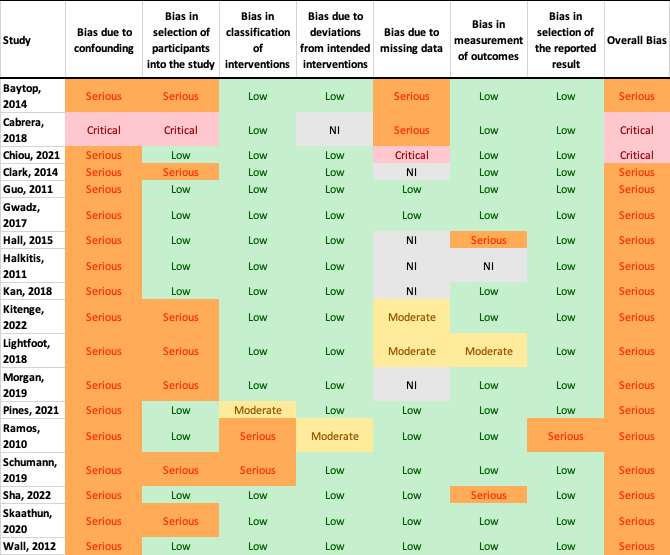


###
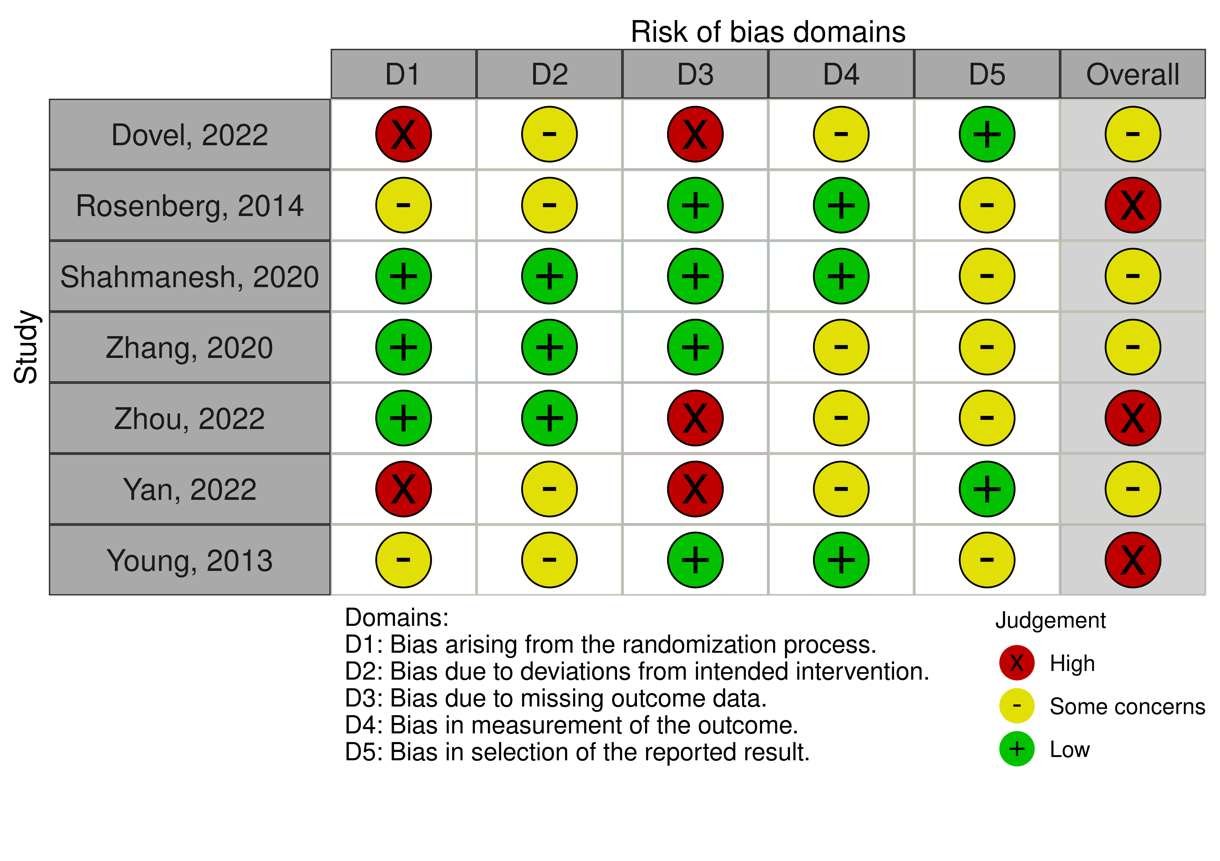
Table S9. Version 2 of the Cochrane risk-of-bias tool for randomised trials (RoB 2)

# Supplementary 3. Forest plots

### Supplementary Figure 1 Proportion of people who tested positive among partners or social contacts of test promoters who linked to care

##
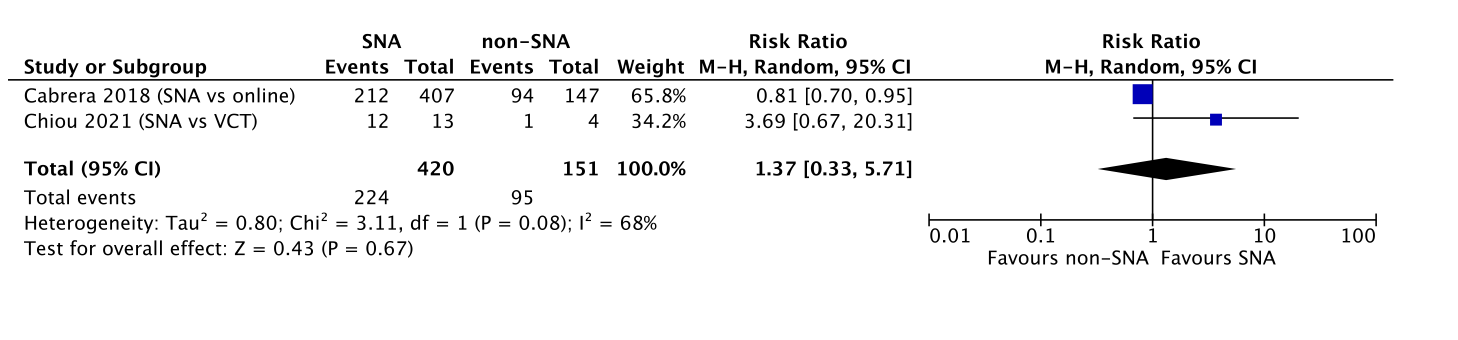


### Supplementary Figure 2 Funnel Plot of uptake of HIV testing among partners or social contacts of test promoters

##
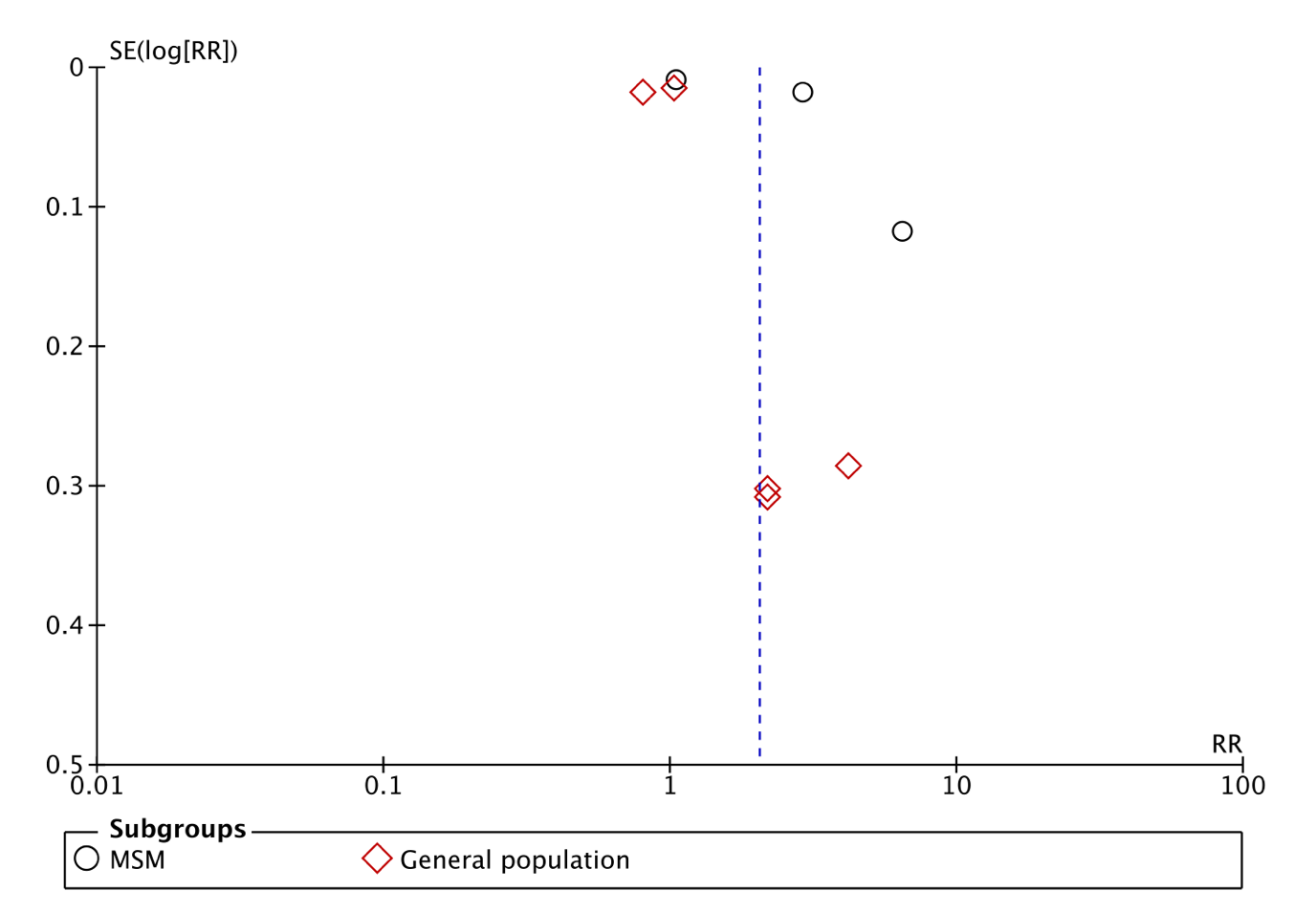


### Supplementary Figure 3 Funnel plot for the proportion of first-time testers among partners or social contacts of test promoters

##
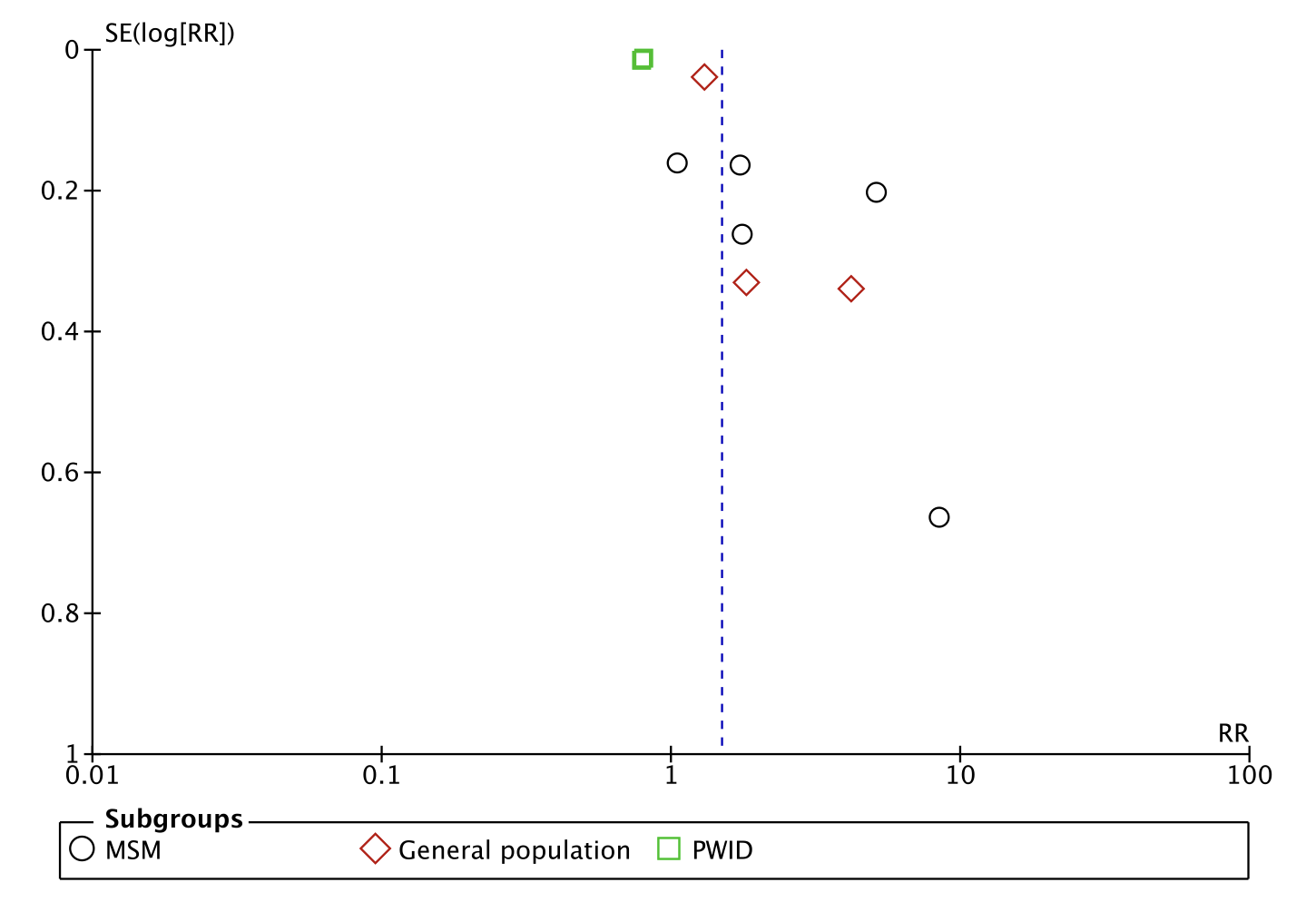


### Supplementary Figure 4 Funnel plot of the proportion of people who tested positive among partners or social contacts of test promoters

##
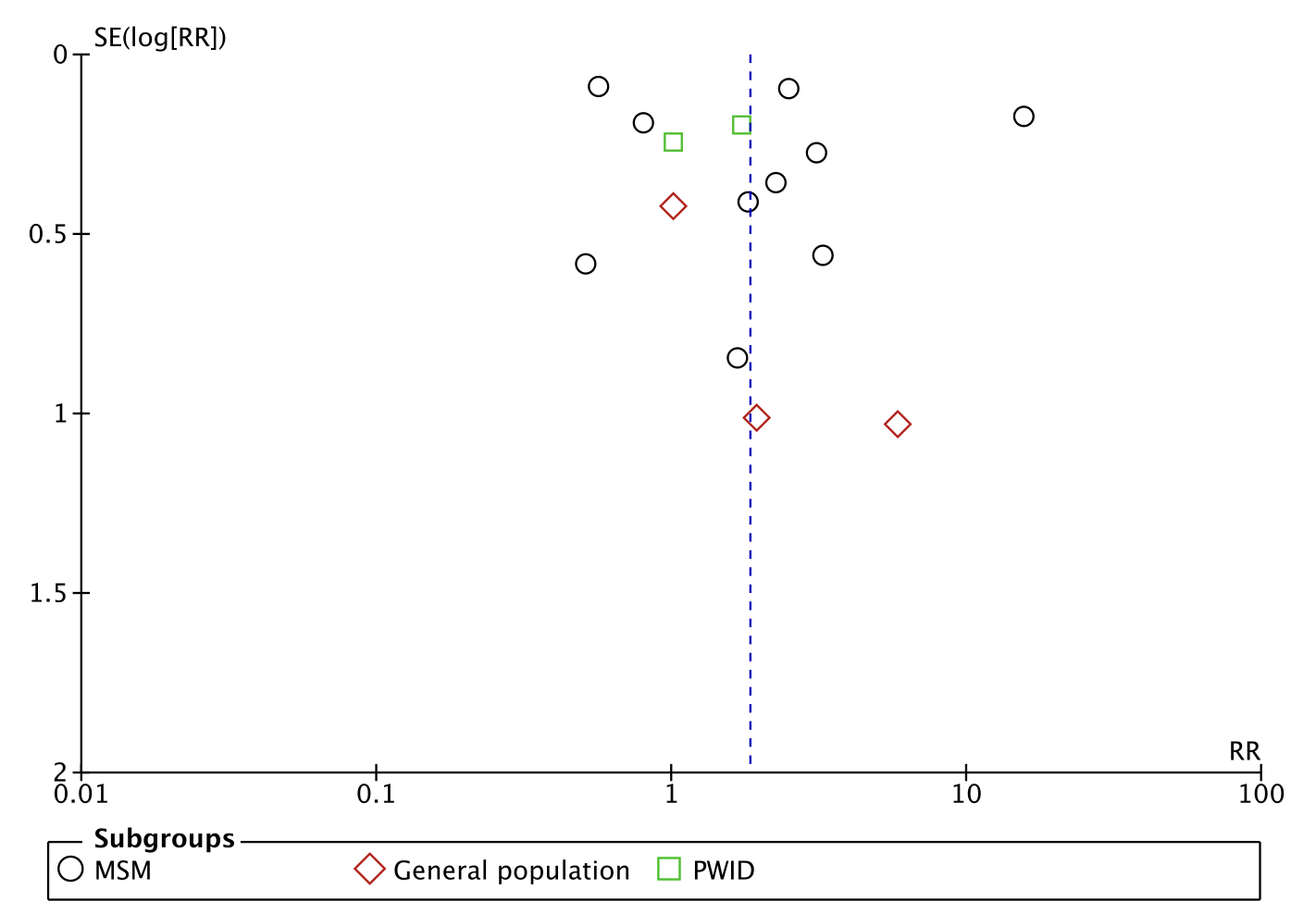


### Supplementary Figure 5 Uptake of SNA for test promoters

##
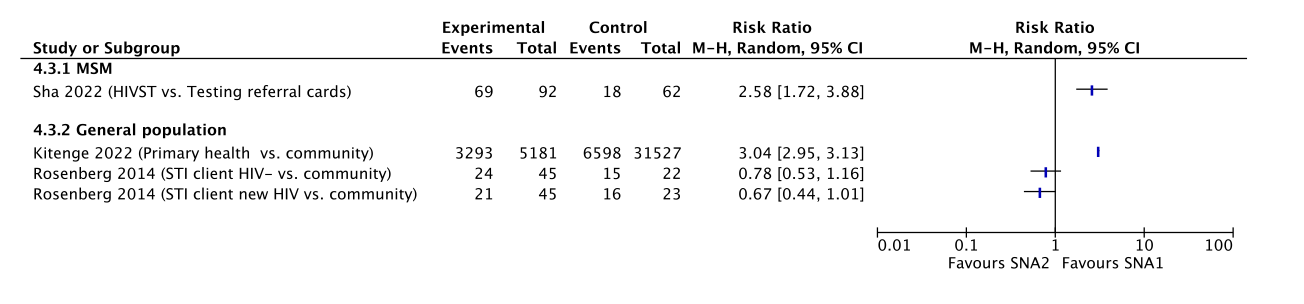


### Supplementary Figure 6 Uptake of HIV testing among partners or social contacts of test promoters

##
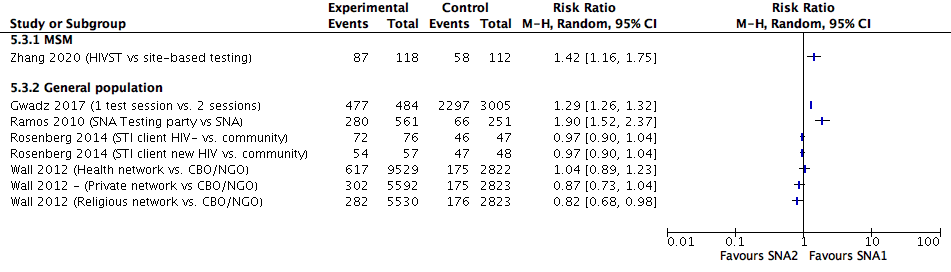


### Supplementary Figure 7 Proportion of first-time testers among partners or social contacts of test promoters

##
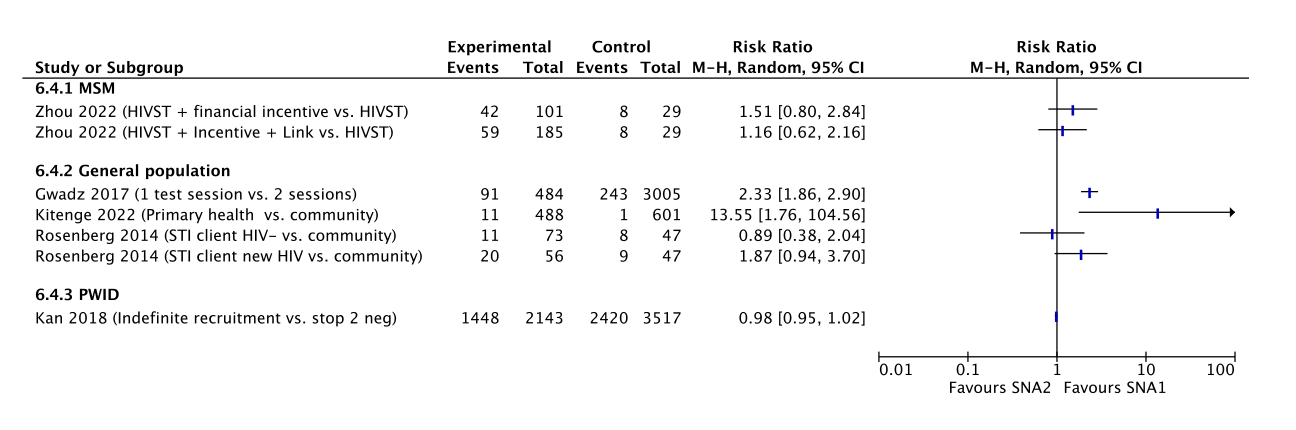


### Supplementary Figure 8 Proportion of people tested positive among partners or social contacts of test promoters

##
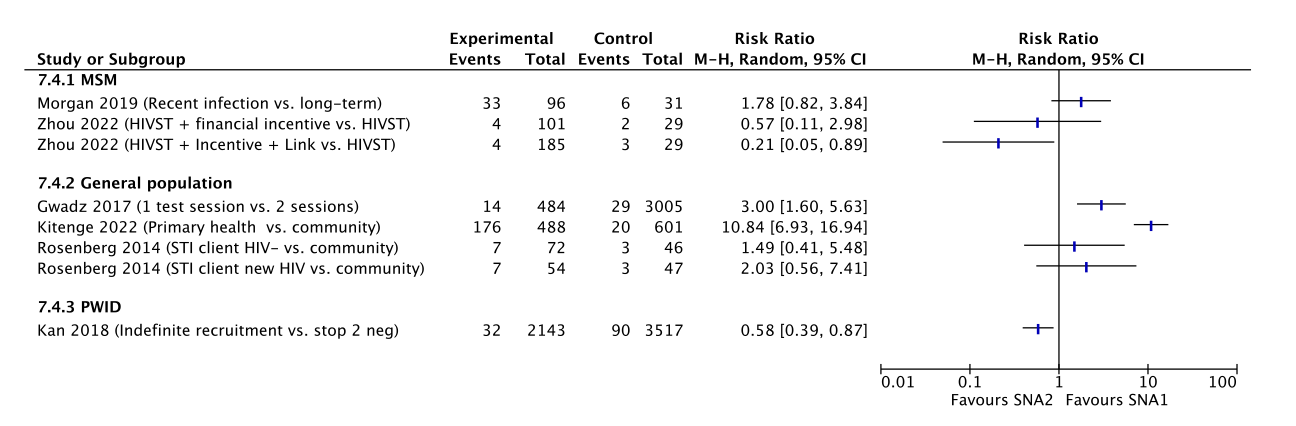


# Supplementary 4 GRADE evidence profile

| **Certainty assessment** | | | | | | | **№ of patients** | | **Effect** | | **Certainty** | **Importance** |
| --- | --- | --- | --- | --- | --- | --- | --- | --- | --- | --- | --- | --- |
| **№ of studies** | **Study design** | **Risk of bias** | **Inconsistency** | **Indirectness** | **Imprecision** | **Other considerations** | **[intervention]** | **[comparison]** | **Relative (95% CI)** | **Absolute (95% CI)** |  |  |
| **1) Proportion of people offered SNA who accepted participating** | | | | | | | | | | | | |
| 0 |  |  |  |  |  |  |  |  | not estimable |  | - | CRITICAL |
| **2) Uptake of HTS among partners/social contacts of test promoters** | | | | | | | | | | | | |
| 6^1,2,3,4,5,6^ | RCT + NRS | serious^a,b,c,d,e,f^ | serious^g^ | not serious | not serious | none | 14098/15515 (90.9%) | 4472/15507 (28.8%) | **RR 2.04** (1.06 to 3.95) | **300 more per 1,000** (from 17 more to 851 more) | ⨁⨁◯◯ Low | CRITICAL |
| **2a) Uptake of HTS among partners/social contacts of test promoters (MSM)** | | | | | | | | | | | | |
| 1^1^ | RCT | serious^a^ | not serious | serious^h^ | serious^i^ | none | 25/57 (43.9%) | 11/55 (20.0%) | **RR 2.19** (1.20 to 4.01) | **238 more per 1,000** (from 40 more to 602 more) | ⨁◯◯◯ Very low | CRITICAL |
| **2b) Uptake of HTS among partners/social contacts of test promoters (MSM)** | | | | | | | | | | | | |
| 3^3,5,6^ | NRS | serious^b,c,f^ | serious^j^ | not serious | not serious | none | 10953/11157 (98.2%) | 4054/14865 (27.3%) | **RR 2.70** (0.63 to 11.55) | **464 more per 1,000** (from 101 fewer to 1,000 more) | ⨁⨁◯◯ Low | CRITICAL |
| **2c) Uptake of HTS among partners/social contacts of test promoters (General population: SNA (single session) vs. Venue-based sampling)** | | | | | | | | | | | | |
| 1^4^ | NRS | serious^d^ | not serious^k^ | not serious | not serious | none | 477/484 (98.6%) | 192/201 (95.5%) | **RR 1.03** (1.00 to 1.07) | **29 more per 1,000** (from 0 fewer to 67 more) | ⨁⨁⨁◯ Moderate | CRITICAL |
| **2d) Uptake of HTS among partners/social contacts of test promoters (General population: SNA (two sessions) vs. Venue-based sampling)** | | | | | | | | | | | | |
| 1^4^ | NRS | serious^d^ | not serious^k^ | not serious | not serious | none | 2297/3005 (76.4%) | 193/202 (95.5%) | **RR 0.80** (0.77 to 0.83) | **191 fewer per 1,000** (from 220 fewer to 162 fewer) | ⨁⨁⨁◯ Moderate | CRITICAL |
| **2e) Uptake of HTS among partners/social contacts of test promoters ('high risk' women: SNA vs. Venue-based sampling)** | | | | | | | | | | | | |
| 1^2^ | NRS | serious^e^ | not serious^k^ | not serious | serious^i^ | none | 66/251 (26.3%) | 11/92 (12.0%) | **RR 2.19** (1.20 to 4.01) | **142 more per 1,000** (from 24 more to 360 more) | ⨁⨁◯◯ Low | CRITICAL |
| **2f) Uptake of HTS among partners/social contacts of test promoters ('high risk' women: Peer-parties vs. Venue-based sampling)** | | | | | | | | | | | | |
| 1^2^ | NRS | serious^e^ | not serious^k^ | not serious | serious^i^ | none | 280/561 (49.9%) | 11/92 (12.0%) | **RR 4.17** (2.38 to 7.31) | **379 more per 1,000** (from 165 more to 754 more) | ⨁⨁◯◯ Low | CRITICAL |
| **3) Proportion of first-time testers among partners/social contacts of test promoters** | | | | | | | | | | | | |
| 8^4,5,7,8,9,10,11,12^ | NRS | serious^l^ | not serious^m^ | not serious | not serious | none | 4850/11153 (43.5%) | 8198/22847 (35.9%) | **RR 1.49** (1.22 to 1.81) | **176 more per 1,000** (from 79 more to 291 more) | ⨁⨁⨁◯ Moderate | IMPORTANT |
| **3a) Proportion of first-time testers among partners/social contacts of test promoters (MSM)** | | | | | | | | | | | | |
| 5^5,9,10,11,12^ | NRS | serious^b,l,n^ | not serious | not serious | not serious | none | 217/772 (28.1%) | 289/3209 (9.0%) | **RR 2.39** (1.25 to 4.60) | **125 more per 1,000** (from 23 more to 324 more) | ⨁⨁⨁◯ Moderate | IMPORTANT |
| **3b) Proportion of first-time testers among partners/social contacts of test promoters (General population)** | | | | | | | | | | | | |
| 2^4,7^ | NRS | serious^d,o^ | not serious | not serious | serious^p^ | none | 765/4721 (16.2%) | 3959/14998 (26.4%) | **RR 2.04** (1.00 to 4.14) | **275 more per 1,000** (from 0 fewer to 829 more) | ⨁⨁◯◯ Low | IMPORTANT |
| **3c) Proportion of first-time testers among partners/social contacts of test promoters (PWID: SNA (indefinite waves) vs. Peer-based testing)** | | | | | | | | | | | | |
| 1^8^ | NRS | serious^q^ | not serious^k^ | not serious | not serious | none | 1448/2143 (67.6%) | 3950/4640 (85.1%) | **RR 0.79** (0.77 to 0.82) | **179 fewer per 1,000** (from 196 fewer to 153 fewer) | ⨁⨁⨁◯ Moderate | IMPORTANT |
| **3d) Proportion of first-time testers among partners/social contacts of test promoters (PWID: SNA (Restricted waves) vs. Peer-based testing)** | | | | | | | | | | | | |
| 1^8^ | NRS | serious^q^ | not serious^k^ | not serious | not serious | none | 2420/3517 (68.8%) | 3950/4640 (85.1%) | **RR 0.81** (0.79 to 0.83) | **162 fewer per 1,000** (from 179 fewer to 145 fewer) | ⨁⨁⨁◯ Moderate | IMPORTANT |
| **4) Percentage of people tested positive for HIV** | | | | | | | | | | | | |
| 13^3,4,5,6,7,8,9,10,11,12,13,14,15^ | NRS | serious^b,c,d,n,o,q,r,s,t^ | serious^u^ | not serious | not serious | none | 803/22871 (3.5%) | 915/70004 (1.3%) | **RR 1.84** (1.01 to 3.35) | **11 more per 1,000** (from 0 fewer to 31 more) | ⨁⨁◯◯ Low | CRITICAL |
| **4a) Percentage of people newly tested positive for HIV (MSM: SNA vs. Venue-based sampling)** | | | | | | | | | | | | |
| 10^3,5,6,9,10,11,12,13,14,15^ | NRS | serious^b,c,n,r,s,t^ | serious^v^ | not serious | not serious | none | 632/12490 (5.1%) | 775/50366 (1.5%) | **RR 1.94** (0.87 to 4.32) | **14 more per 1,000** (from 2 fewer to 51 more) | ⨁⨁◯◯ Low | CRITICAL |
| **4b) Percentage of people newly tested positive for HIV (General population: SNA vs. non-SNA)** | | | | | | | | | | | | |
| 2^4,7^ | NRS | serious^d,o^ | not serious^w^ | not serious | serious^i^ | none | 49/4721 (1.0%) | 72/14998 (0.5%) | **RR 1.63** (0.60 to 4.43) | **3 more per 1,000** (from 2 fewer to 16 more) | ⨁⨁◯◯ Low | CRITICAL |
| **4c) Percentage of people newly tested positive for HIV (PWID: SNA (indefinite waves) vs. non-SNA)** | | | | | | | | | | | | |
| 1^8^ | NRS | serious^q^ | not serious^k^ | not serious | serious^i^ | none | 32/2143 (1.5%) | 34/2320 (1.5%) | **RR 1.02** (0.63 to 1.65) | **0 fewer per 1,000** (from 5 fewer to 10 more) | ⨁⨁◯◯ Low | CRITICAL |
| **4d) Percentage of people newly tested positive for HIV (PWID: SNA (restricted waves) vs. non-SNA)** | | | | | | | | | | | | |
| 1^8^ | NRS | serious^q^ | not serious^k^ | not serious | not serious^i^ | none | 90/3517 (2.6%) | 34/2320 (1.5%) | **RR 1.75** (1.18 to 2.58) | **11 more per 1,000** (from 3 more to 23 more) | ⨁⨁⨁◯ Moderate | CRITICAL |
| **5) Baseline CD4 count or viral load among people diagnosed with HIV** | | | | | | | | | | | | |
| 0 |  |  |  |  |  |  |  |  | not estimable |  | - | IMPORTANT |
| **6) Proportion tested positive who linked to care (MSM)** | | | | | | | | | | | | |
| 2^5,6^ | NRS | serious^b,f^ | not serious^k^ | not serious | serious^i^ | none | 224/420 (53.3%) | 95/151 (62.9%) | **RR 1.37** (0.33 to 5.71) | **233 more per 1,000** (from 422 fewer to 1,000 more) | ⨁⨁◯◯ Low | IMPORTANT |
| **7) Identifying people with HIV who are not engaged in care (e.g. not on ART, or not virally suppressed).** | | | | | | | | | | | | |
| 0 |  |  |  |  |  |  |  |  | not estimable |  | - | IMPORTANT |

**CI:** confidence interval; **NRS**: non-randomised comparator studies; **RCT**: randomised controlled trials; **RR:** risk ratio

#### Explanations

a. Risk of bias: Young 2013 (Cluster-RCT). Downgraded because people delivering SNA were aware of the assigned intervention (no blinding). No pre-specified analysis plan.

b. Risk of bias: Chiou 2021 (Quasi-experimental). Downgraded because though propensity matching was used in the final analyses, data were based on self-report.

c. Risk of bias: Pines 2021 (Quasi-experimental). Downgraded because though Poisson regression analysis was used, data were based on self-report. May be some misclassification of HIV status during eligibility screening (due to self-report). The authors noted the difficulty in distinguishing "new HIV diagnosis" from previously diagnosed.

d. Risk of bias: Gwadz 2017 (Non-randomised parallel study design). Downgraded because did not control for confounders, and had 25% loss of participants in session 2 of RDS-CTT (confidential, two-session testing) group.

e. Risk of bias: Ramos 2010 (Quasi-experimental). As there were insufficient numbers of staff to run the three phases concurrently, the phases were run in sequential six- month intervals. Downgraded because women provided demographic characteristics but this was not reported in the manuscript (so we could not assess for similarities/differences at baseline between the two groups). No further information on participants except they were 'at-risk women'. No statistical adjustment for confounding.

f. Risk of bias: Cabrera 2018 (Quasi-experimental). Downgraded because of self-reported data.

g. Inconsistency: Downgraded because of high heterogeneity (I^2=99%, p<0.00001), not explainable by subgroup analyses according to population or study design.

h. Study randomises people to either discuss about HIV or "general health" to Facebook social contacts.

i. Imprecision: Downgraded because of <400 events.

j. Inconsistency. Although both effect sizes favoured SNA, they had non-overlapping confidence intervals. Chiou 2021 was conducted in Taiwan and reported RR 6.47 (95% CI: 5.14-8.15). Pines 2021 was conducted in Mexico and reported RR 1.05 (95% CI: 1.03-1.07).

k. Inconsistency: Unable to assess because of a single study.

l. Risk of bias: All studies relied on self-reported data for being a first-time tester.

m. Inconsistency: There was large heterogeneity (I^2=97%, p<0.00001), but this was explained by the population type and the comparator. SNA approaches probably increase the proportion of first-time testers for MSM and may increase the proportion of first-time testers for the general population. However, SNA approaches (compared to peer-testing) for PWID may decrease the proportion of first-time testers.

n. Risk of bias: Hall 2015 (Quasi-experimental). Downgraded because of self-reported data. There is no information about missing data or loss to follow up.

o. Risk of bias: Schumann 2019 (Quasi-experimental) Downgraded because it did not control for confounders statistically. The study's eligibility was for 'at-risk' individuals, but inclusion criteria were not specified. Some recruiters recruited more than 20 social network clients, but pre-intervention, the cap was 20. There is no further information on missing data and how it was handled.

p. Though both studies found a significantly higher proportion of first-time testers among social contacts of index clients for the SNA approach (Gwadz: 4.21, 95% CI: 2.58-6.86; Schumann: 1.30, 95% CI: 1.20-1.40), the pooled risk ratio was 2.28, 95% CI: 0.70-7.37. A decision to choose SNA may change if the true value was either the lower or higher bounds estimate.

q. Risk of bias: Kan 2018 (Quasi-experimental). Downgraded because of self-reported data.

r. Risk of bias: Skaathun 2020 (Cohort). Downgraded because the three methods of testing occurred at different times: Routine hospital-based testing (2011-2016); Risk network strategy (2013-2016); RDS (2013-2014).

s. Risk of bias: Guo 2011 (Quasi-experimental). Downgraded because of self-reported data.

t. Risk of bias: Halkitis 2011 (Quasi-experimental). Downgraded because of self-reported data.

u. Inconsistency: Downgraded because of high heterogeneity (I^2 =95%, p<0.00001), partly explained by population type and type of SNA. Note that heterogeneity remained high within the MSM studies (I^2=97%, p<0.00001) with non-overlapping confidence intervals.

v. Four of six studies reported a significantly higher percentage of people who tested positive for HIV using SNA. Guo reported a higher (but not statistically significant) risk ratio (1.68, 95% CI: 0.32-8.85), whilst Pines reported a lower (but not statistically significant) risk ratio (0.80, 95% CI: 0.55-1.18).

w. Inconsistency: One study (Schumann) reported no difference in HIV test positivity comparing SNA vs. non-SNA (RR 1.02, 95% CI: 0.44-2.33), whilst one study reported significantly higher HIV test positivity for those in the SNA group (RR 5.83, 95% CI: 1.33-25.49). However, we did not downgrade as there were overlapping 95% confidence intervals.

#### References

1.Young, S. D., Cumberland, W. G., Lee, S. J., Jaganath, D., Szekeres, G., Coates, T.. Social networking technologies as an emerging tool for HIV prevention: a cluster randomised trial. Annals of Internal Medicine; 2013.

2.Ramos, R. L., Ferreira-Pinto, J. B., Rusch, M. L. A., Ramos, M. E.. Pasa la Voz (spread the word): Using women’s social networks for HIV education and testing. Public Health Reports; 2010.

3.Pines, H. A., Semple, S. J., Magis-Rodriguez, C., Harvey-Vera, A., Strathdee, S. A., Patrick, R., Rangel, G., Patterson, T. L.. A comparison of the effectiveness of respondent-driven and venue-based sampling for identifying undiagnosed HIV infection among cisgender men who have sex with men and transgender women in Tijuana, Mexico. Journal of the International AIDS Society; 2021.

4.Gwadz, M., Cleland, C. M., Perlman, D. C., Hagan, H., Jenness, S. M., Leonard, N. R., Ritchie, A. S., Kutnick, A.. Public health benefit of peer-referral strategies for detecting undiagnosed HIV infection among high-risk heterosexuals in New York City. Journal of Acquired Immune Deficiency Syndromes; 2017.

5.Chiou, P. Y., Ko, N. Y., Chien, C. Y.. Mobile HIV testing through social networking platforms: Comparative study. Journal of Medical Internet Research; 2021.

6.AJ, Cabrera,Oliva, S, Lungo, C, Castellan, C., Palma. Can online interventions enhance HIV case-finding and linkages to care? Comparing offline and online monitoring data from a combination prevention program with MSM and transgender women in Central America.. Abstract Book AIDS 2018. 22nd International AIDS Conference; 2018 Jul 23-27 Amsterdam, The Netherlands: International AIDS Society (IAS); 2018. p. 459.; 2018.

7.Schumann, Casey, Kahn, Danielle, Broaddus, Michelle, Dougherty, Jacob, Elderbrook, Megan, Vergeront, James, Westergaard, Ryan. Implementing a standardised social networks testing strategy in a low HIV prevalence jurisdiction. AIDS and Behavior; 2019.

8.Kan, M., Garfinkel, D. B., Samoylova, O., Gray, R. P., Little, K. M.. Social network methods for HIV case-finding among people who inject drugs in Tajikistan. J Int AIDS Soc; 2018.

9.Hall, Grace, Li, Keala, Wilton, Leo, Wheeler, Darrell, Fogel, Jessica, Wang, Lei, Koblin, Beryl. A comparison of referred sexual partners to their community recruited counterparts in The BROTHERS Project (HPTN 061). AIDS and Behavior; 2015.

10.Lightfoot, M. A., Campbell, C. K., Moss, N., Treves-Kagan, S., Agnew, E., Kang Dufour, M. S., Scott, H., Sa'id, A. M., Lippman, S. A.. Using a Social Network Strategy to Distribute HIV Self-Test Kits to African American and Latino MSM. J Acquir Immune Defic Syndr; Sep 1 2018.

11.Clark, J. L., Konda, K. A., Silva-Santisteban, A., Peinado, J., Lama, J. R., Kusunoki, L., Perez-Brumer, A., Pun, M., Cabello, R., Sebastian, J. L., Suarez-Ognio, L., Sanchez, J.. Sampling methodologies for epidemiologic surveillance of men who have sex with men and transgender women in Latin America: an empiric comparison of convenience sampling, time space sampling, and respondent driven sampling. AIDS Behav; Dec 2014.

12.Baytop, C., Royal, S., Hubbard McCree, D., Simmons, R., Tregerman, R., Robinson, C., Johnson, W. D., McLaughlin, M., Price, C.. Comparison of strategies to increase HIV testing among African-American gay, bisexual, and other men who have sex with men in Washington, DC. AIDS Care; 2014.

13.Skaathun, B., Pho, M. T., Pollack, H. A., Friedman, S. R., McNulty, M. C., Friedman, E. E., Schmitt, J., Pitrak, D., Schneider, J. A.. Comparison of effectiveness and cost for different HIV screening strategies implemented at large urban medical centre in the United States. Journal of the International AIDS Society; 2020.

14.Guo, Y., Li, X., Fang, X., Lin, X., Song, Y., Jiang, S., Stanton, B. A comparison of four sampling methods among men having sex with men in China: Implications for HIV/STD surveillance and prevention. AIDS Care - Psychological and Socio-Medical Aspects of AIDS/HIV; 2011.

15.Halkitis, P. N., Kupprat, S. A., McCree, D. H., Simons, S. M., Jabouin, R., Hampton, M. C., Gillen, S.. Evaluation of the relative effectiveness of three HIV testing strategies targeting African American men who have sex with men (MSM) in New York City. Annals of Behavioral Medicine; 2011.

# Supplementary 5

### Table S10 Description of types of SNA models

| **Study identifier** | **Study design** | **Country** | **Year** | **Study population** | **Types of SNA** |
| --- | --- | --- | --- | --- | --- |
| Baytop 2014 | Quasi-experimental | USA | 2008-2010 | MSM | **1) SNA**  - Test promoters encouraged men in their social and sexual networks for HIV testing  - recruited individuals who were high risk for HIV (i.e. condomless sex or shared drug injection equipment within the preceding 6 months) were invited to be test promoters  **2) Venue-based sampling**  - HIV testing offered via mobile testing van or pre-arranged private spaces  **Extra notes:**  - Test promoters received $20 per person tested  - Recruited individuals also received $20 for HIV testing (as per standard procedure at the HIV testing site)  - Multiple waves |
| Cabrera 2018 | Quasi-experimental | Guatemala, El Salvador, Honduras, Panama | 2016-2017 | MSM + TGW | **1) SNA**  - peers referred individuals from their social network for HIV testing  **2) Online recruitment**  - “Cyber-educators” were trained to use social media platforms (Facebook, Whatsapp) to generate demand for and refer to HIV testing among at-risk MSM and TGW with emphasis on “hidden” populations  **Extra info:**  - no financial incentives for recruitment  - unclear regarding the number of waves |
| Chiou 2021 | Quasi-experimental | Taiwan | 2018 | MSM | **1) SNA**  - social network members received free and anonymous rapid HIV tests at a time and place of their choosing  - ad in gay dating apps and Facebook  **2) VCT**  - free and anonymous rapid HIV testing ad on the hospital website  - screening station near the entrance of gay village between 6-10 pm every Friday and Saturday in Taipei  **Extra info:**  **-** No financial incentives for recruitment  - Single wave |
| Clark 2014 | Quasi-experimental | Peru | 2011 | MSM + TGW | **1) SNA**  - 24 test promoters (mix of heterosexual/bisexual MSM, homosexual MSM, TGW, non-transgender male sex workers) given 5 recruitment coupons to distribute to social networks  **2) VBS**  - mobile testing unit in venues where a minimum of 20 MSM/TGW observed  **Extra info:**  - Test promoters were given $6 for each recruit enrolled  - Unclear regarding the number of waves |
| Guo 2011 | Quasi-experimental | China | 2009 | MSM | **1) SNA**  - ‘refer friends’ to participate  **2) Peer outreach**  - Three MSM hired to approach other MSM  **3) Internet recruitment**  - announcements on local gay websites  **4) Venue-based sampling**  - distribution of study information in MSM-frequented venues  **Extra info:**  - No financial incentives  - Single wave |
| Gwadz 2017 | Non-randomised parallel study design | USA | 2012-2015 | General population | **1) SNA + Requirement to attend two-sessions**  - first session: training on peer recruitment (how to encourage/offer HIV testing)  - second session: providing rapid HIV testing on site  **2) SNA + Single-session testing**  - training on peer recruitment and laboratory based-testing at the first visit  **3) Venue-based sampling**  - methods that identify days and times when the target population gathers at specific venues, constructing a sampling frame of venue/day-time units and then randomly visits and consistently engages members of the target population.  **Extra info:**  - Test promoters received compensation for recruitment of peers ($15/eligible peer)  - Test promoters encouraged to recruit 3-6 social contacts  - Multiple waves: Social contacts encouraged to recruit further 3-5 social contacts |
| Halkitis 2011 | Quasi-experimental | USA | 2008-2009 | MSM | **1) SNA**  - Test promoters were assisted in developing a plan for recruitment and coached on how to engage their social network for HIV testing  **2) Venue-based sampling**  - Mobile van in locations frequented by African American MSM  - Testing available at least once per week for 4 hours per event  **3) Partner services**  **-** no further details provided in manuscript  **Extra info**  - Test promoters received $10 for each social network member tested  - Single wave |
| Hall 2015 | Quasi-experimental | USA | 2009-2010 | MSM (African American) | **1) SNA**  - Test promoters could recruit up to 5 sexual partners who were African American MSM  2) **Community recruited**  - ‘Recruited men directly from the community’  **Extra info:**  **-** Test promoters received $5 (Boston) up to $20 (Los Angeles) per social network member tested  - Multiple waves |
| Kan 2018 | Quasi-experimental | Tajikistan | 2016-2017 | PWID | **1) SNA with unrestricted waves**  - Each tested social contact also received additional coupons to recruit members of their social network  **2) SNA with restricted waves**  - If two successive individuals were recruited who were HIV-negative, no coupons were provided for further recruitment  **3) Peer-based active case finding**  - Living with HIV, or former or current PWID recruited peers through direct outreach  **Extra info:**  - Social contacts received $3 after testing and 3 recruitment coupons, instructions on who to recruit and how to do so  - Recruiters received an additional non-cash incentive (equivalent to $1.50) for each additional social contact who redeemed the coupon and underwent testing  - Multiple waves (as described above) |
| Kitenge 2022 | Cross-sectional | South Africa | 2018-2020 | General population | **Choosing test promoters for SNA (HIVST)**  - Attendees of primary health clinics  - Attendees of community-based testing sites for peer distribution and/or self-testing  Extra info:  - Test promoters were given 2-5 HIVST kits for distribution and/or self-testing  - HIV counsellors and community health workers provided a brief explanation of how to use the test kits and what to explain to social contacts before handing out HIVST kits  - Instructed to distribute the HIVST kits to their sexual partner, family members or anyone in their social network  - Single wave |
| Lightfoot 2018 | Quasi-experimental | USA | 2016-2017 | MSM/TGW | **1) SNA**  - Test promoters identified from HIV-related support groups, local gay bars, online social networking and dating apps, community-based organisations, and word of mouth  - Test promoters underwent training on HIV basics, HIVST kit use, resources for confirmatory testing and treatment, and data collection procedures  - Test promoters were given 5 HIVST kits  2) **Standard care**  - Community-based testing that targeted MSM and TGW, especially African Americans and Latinx  - Some included mobile and venue-based testing at various sites, including bars and bathhouses  **Extra info:**  - Test promoters received $100 for 3-hour training and $150 after distributing 5 HIVST kits  - Single wave |
| Morgan 2019 | Quasi-experimental | USA | 2013-2016 | MSM/TGW | **Choosing test promoters for SNA**  1) Recently infected  - Test promoters had laboratory evidence of acute infection or documented seroconversion in the last 9 months  - Test promoters recruited social network members who were sexual or drug-using partners  2) Long-term infected  - Test promoters were newly diagnosed but did not fit the criteria above  Extra info:  - Received $20 for each social contact tested  - Multiple waves: recruitment stopped after 2 waves (if no social contact tested HIV positive) |
| Pines | Quasi-experimental | Mexico | 2015-2018 | MSM/TGW | **1) SNA**  - Test promoters received three to six coupons  - Social network members also received three to six coupons when they presented for testing  - Staff helped test promoters develop peer outreach plans  **2) Venue-based testing**  - Recruited from 34 physical venues (e.g. bars/clubs, public spaces), one gay-dating app and 6 special events (e.g. Gay Pride)  - To incentivise testing, persons were invited to spin a wheel that offered non-coercive prizes (e.g. flavoured lubricants)  **Extra info:**  - Received $5 for every social network member referred  - Multiple waves |
| Ramos 2010 | Quasi-experimental | Mexico | 2005-2007 | Women | **1) Direct outreach**  - Community leaders encouraged other women to access free HIV testing at health service organisations  **2) SNA**  - Test promoters were selected by community leaders and encouraged to identify other women in their networks who they believed were at risk for HIV  – Offered testing by test promoters via referral to a testing centre.  **3) Peer-organised testing parties (using social networks to organise HIV testing parties)**  - Test promoters invited individuals to their homes to provide a safe, comfortable environment to talk about HIV and receive rapid HIV testing (conducted by healthcare workers)  **Extra info:**  - No financial incentives  - Single wave |
| Rosenberg 2014 | Matched cross-sectional | Malawi | 2010-2012 | STI clinic attendees | **Choosing test promoters for SNA**  - Clients newly diagnosed with HIV among STI clinic patients with STI syndromes  - HIV-negative STI clinic clients with STI syndromes- Community controls (matched by age, gender and geographical area)  **Extra info:**  - Test promoters were asked to recruit up to 5 social contacts who they thought would benefit (not restricted to sexual partners or family members)  - Contacts were also offered blood pressure screening, health promotion discussion on cardiovascular disease, diabetes, clean water and hygiene, family planning, STIs, or malaria, and received $5 for transport reimbursement.  - Single wave |
| Schumann 2019 | Quasi-experimental | USA | 2013-2015 | General population | **1) SNA**  - Test promoters underwent program orientation and a coaching session (taught techniques for talking with selected social network members about how to access HIV testing services)  - Test promoters could recruit up to 20 social network members  2) **VCT**  - All recruitment methods other than SNA  **Extra info:**  - $10 given to test promoter + social network member for each HIV testing event  - Single wave |
| Sha 2022 | Quasi-experimental | China | 2019 | MSM | **1) SNA with HIVST**  - Test promoters could distribute HIV/syphilis dual test kits to people within their social network  **2) SNA with HIV Testing invitation cards**  - Test promoters could send (up to 5) HIV testing invitation cards to their social networks for free facility-based tests  **Extra info:**  - Test promoters are encouraged to distribute within a month  - Social contacts are encouraged to upload a photo verification of their results  - Test promoters and their social contacts received incentives ($3 when the social contact uploaded results)  - Single wave |
| Shahmanesh | Cluster-RCT | South Africa | 2019 | General population | **1) Incentivised SNA**  - Test promoters could distribute up to 5 HIVST kits to 18-30 year olds within their social network  **2) Standard of care**  - Peer navigators distributed clinic referral slips, and information about HIV and PrEP to 18-30 year olds  **3) Peer navigator distribution**  - Peer navigator distributed 2 HIVST kits  **Extra info:**  - Test promoters received $1.50 for each recipient who distributed further packs  - Multiple waves |
| Skaathun 2020 | Cohort | USA | 2011-2016 | MSM (African American) | **1) SNA**  **-** Test promoters were given up to six vouchers to recruit young African American MSM (16-29 years old)  **2) Partner services among networks of recently HIV infected (<9 months)**  - Contact tracing of drug-using partners  **3) Routine screening**  - Included screening in the emergency department, inpatient and outpatient settings  **Extra info:**  - Test promoters (SNA) received $20 for each enrolled social network member  - Unclear how many waves, likely single wave |
| Wall 2012 | Cohort | Zambia | 2004-2005 | General population | **Choosing seeds for SNA**  - Faith-based religious networks- Health  - Private  - Community-based/non-governmental organisations  **Extra info:**  **-** Test promoters provided with 4-day training in HIV/AIDS advocacy/outreach, social networking, free couples VCT promotions and observation of successful door-to-door promotional strategies  - Test promoters received $0.21 per invitation issued and an additional $4.20 per couple attending CVCT. (later changed to $0.11 and $5.25, respectively)  - Single wave |
| Young | Cluster-RCT | USA | 2010-2011 | MSM | **Using social network technology (Facebook)**  1) Intervention: Test promoters were instructed to communicate about HIV testing and prevention  2) Control: individuals were instructed to communicate about “general health” (the importance of exercising, healthy eating, and maintaining a low-stress lifestyle)  **Extra info:**  - No financial incentives  - Single wave |
| Zhang 2020 | RCT | China | 2018 | MSM | **1) SNA (HIVST)**  - Test promoters received 2 free HIVST kits and could receive 2-4 kits delivered through express mail every 3 months for 1 year, in addition to access to site-based HIV testing. Encouraged to distribute kits to their sexual partners.  **2) SNA (site-based testing)**  - Access to site-based HIV testing (local hospitals, free HIV testing and counselling clinics at local CDC, and gay-friendly CBOs)  Extra info:  - No financial incentives  - Single wave |
| Zhou 2022 | RCT | China | 2019-2020 | MSM | **1) SNA (HIVST)**  - Test promoters can order up to five HIVST kits  - Paid refundable deposit for each kit ($15)  - Encouraged to upload a photo of test results via QR code  **2) SNA (HIVST) + monetary incentive**  - As above + receive $3 for each upload of test results from social contacts (maximum of $15)  **3) SNA (HIVST) + monetary incentive + peer referral link**  - As above + peer referral link (where social contact was not given an HIVST kit directly but had to order it themselves)  - Received monetary incentive for up to 10 social contacts (maximum of $30) – up to 5 from direct distribution of HIVST kits and up to 5 from a peer referral link  **Extra info:**  - Financial incentives (as described above)  - Single wave |
| Zulliger 2017 | Cost-utility analysis | USA | 2013-2014 | MSM | **1) SNA**  - encouraged peers to test for HIV  **2) Venue-based testing**  - public and private locations attended by MSM  **3) Couples VCT**  - two or more persons who were, or were planning to be, in a sexual relationship received HIV testing and counselling together  **Extra info:**  - No financial incentives  - Unclear how many waves, likely single |

*Notes:* All currencies are presented as US dollars

# Supplementary 6 Resource Use

### Table S11 Costs for SNA vs. non-SNA

| Study | Setting | Costs included | Results |
| --- | --- | --- | --- |
| Skaathun | USA, MSM | Healthcare provider perspective (USD 2016) Personnel time, HIV testing, training, materials, overheads | SNA: $580,260  Partner services: $491,3247 Hospital-based testing: $2,767,481 |
| Pines | Mexico, MSM/TGW | Healthcare provider perspective (USD 2017)  Personnel time, advertisements, equipment, materials, incentives, transportation, overheads | SNA: $189,823 Venue-based testing: $313,871 |
| Zulliger | USA, MSM | Societal perspective (USD 2014) Personnel time, materials, overheads, testing incentives, costs incurred by clients (financial and time costs for travel and visit) | SNA: $33,897 Venue-based testing: $3,085,500 |
| Ramos | Mexico, General population | Personnel time per person tested | SNA: 6.18 hours  SNA with Testing Parties: 3.68 hours  One-on-one outreach: 22.7 hours |

## MSM = men who have sex with men, SNA = social network-based approaches, TGW = transgender women

### Table S12 Costs for types of SNA

| **Study** | **Setting** | **Costs included** | **Results** |
| --- | --- | --- | --- |
| Shahmanesh | South Africa, General population | Healthcare provider perspective (USD, currency year unknown, likely 2019)  Capital costs, training for peer navigators, personnel costs, supplies, transport, HIVST kits, and financial incentives. | SNA: $57,055  Peer-navigator distribution HIVST: $82,311  Standard of care (Peer-navigator discussion): $69,757 |
| Zhou | China, MSM | Healthcare provider perspective (USD 2020)  Capital costs, personnel costs, consumables, financial incentives | SNA + Financial incentive (FI): $6,219  SNA + FI + Peer referral link: $7,688  SNA + No FI: $5,578 |
| Sha | China, MSM | Healthcare provider perspective (USD 2020)  Capital costs, personnel costs, consumables | SNA + HIVST: $16,692  SNA + testing referral cards: $9,408 |

FI = financial incentive, HIVST = HIV self-testing, MSM = men who have sex with men, SNA = social network-based approaches, USD = United States dollars

Resource use comparing types of SNA identified strategies that were probably cheaper compared to other types of SNA. This was based on three studies (2 for MSM^1, 2^ and 1 for the general population^3^). For MSM in China, one study^2^ reported providing financial incentives is probably cheaper per person tested but not per person diagnosed (*Moderate certainty*). Another study of MSM in China reported providing HIVST kits to distribute compared to HIV testing referral cards is probably cheaper per person tested (*Moderate certainty*). For the general population in South Africa^3^, SNA with incentives was probably cheaper per HIVST kit distributed than peer distribution alone (*Moderate certainty*). Shahmanesh et al.^3^ reported the total programme cost for the general population from a healthcare provider perspective. Capital costs included equipment (laptops for nurse and administrator, study phones and tablets for peer navigators) and training for the peer navigators (staff costs to train and supervise the peer navigators and external training). Recurrent costs included personnel costs (the peer navigators stipend, calculated per hour of work and staff to supervise the peer navigators), supplies (packs, health promotion material, referral slips, data for tablet connectivity and protective clothing), transport (including delivering packs to peer navigators in the field), Oraquick HIVST test kits, RDS incentives and other (mobile phone air time). The total cost for incentivised SNA was $57,055 (USD, currency year unknown), peer distribution was $82,311, and standard-of-care was $69,757. Zhou et al.^2^ reported the total programme costs for MSM from a healthcare provider perspective (including fixed capital costs, fixed costs for staff, fixed consumable costs, variable staff costs, and variable consumable costs) for SNA with financial incentive was $6219 (USD 2020), SNA with financial incentive and peer-referral was $7688, and no financial incentive was $5578. Sha et al.^1^ reported the total programme costs for MSM from a healthcare provider perspective (including fixed costs consisting of building rent, office equipment, and personnel; variable costs consisting of consumables, telephone bills, and transport) for SNA was $16,692 (USD 2020) for SNA with HIVST kits and $9408 for SNA with testing referral cards.

### Table S13 Cost-effectiveness for types of SNA

| Study | Setting | Cost per person tested | Cost per person diagnosed |
| --- | --- | --- | --- |
| Shahmanesh | South Africa, General population | SNA: $36 (per kit distributed)Peer-navigator distribution HIVST: $56Standard of care: $64 |  |
| Zhou | China, MSM | SNA + Financial incentive (FI): $62SNA + FI + Peer referral link: $42SNA + No FI: $96 | SNA + Financial incentive (FI): $1,555SNA + FI + Peer referral link: $1,538SNA + No FI: $930 |
| Sha | China, MSM | SNA + HIVST: $120SNA + testing referral cards: $9,408 | SNA + HIVST: $2,348SNA + testing referral cards: NA |

# Supplementary 7 Qualitative data

For FSW, SNA strategies were probably acceptable and varied according to the nature of their emotional or social relationship with their social network, with greater acceptability for their main partner (Moderate confidence). For adolescents and young women, SNA was probably acceptable as they could avoid harm, discuss about HIV and the importance of testing with their partners, and feel empowered in the process (Moderate confidence). For youth, peer-to-peer SNA was probably acceptable as they felt comfortable sharing sexual health issues they would not share with adults, but some youth raised concerns about receiving sexual health information from their peers who were seen as non-professionals (Moderate confidence). For MSM/TGW, SNA was probably acceptable when their social contacts were open-minded, meaning they did not see SNA as an invitation to sex, and acceptability varied depending on how SNA was framed, e.g. part of the research project, gradually incorporating testing mentions into discussions about sexual health, or using the HIVST kits to facilitate joint testing (Moderate confidence). For the general population, SNA was probably acceptable, especially when distributing HIVST kits, as it could provide earlier HIV identification, was convenient, avoided STI clinic-related stigma, and was empowering, but some raised concerns about the accuracy and legitimacy of the kit, and not receiving HIV results from a doctor (Moderate confidence). In one general population study, we also found that men may trust their male peers more than their female partners when it comes to discussions about HIV testing since they have already discussed their sexual behaviours with each other (Low confidence).

### Table S14 Summary of Qualitative Findings

| # | Summarised review finding | GRADE-CERQual assessment of confidence | Explanation of GRADE-CERQual assessment | References |
| --- | --- | --- | --- | --- |
| 1. Acceptability | | | | |
| 1 | Adolescent and young women: SNA was acceptable as they could avoid harm, discuss about HIV and the importance of testing with their partners, and feel empowered in the process. | Moderate confidence | No/Very minor concerns regarding methodological limitations, Moderate concerns regarding coherence, Minor concerns regarding adequacy, and Moderate concerns regarding relevance | Tembo et al. 2020; Wango et al. 2021; |
| 2 | Youth: Peer-to-peer SNA was acceptable as they felt comfortable sharing sexual health issues they would not share with adults, but some youth raised concerns about receiving sexual health information from their peers who were seen as non-professionals. | Moderate confidence | No/Very minor concerns regarding methodological limitations, No/Very minor concerns regarding coherence, Serious concerns regarding adequacy, and Minor concerns regarding relevance | Adeagbo et al. 2022; |
| 3 | MSM/TGW: SNA was acceptable when their social contacts were open-minded, meaning they did not see SNA as an invitation to sex, and acceptability varied depending on how SNA was framed, e.g. part of the research project, gradually incorporating testing mentions into discussions about sexual health, or using the HIVST kits to facilitate joint testing. | Moderate confidence | Minor concerns regarding methodological limitations, No/Very minor concerns regarding coherence, No/Very minor concerns regarding adequacy, and Moderate concerns regarding relevance | Frasca et al. 2014; Iribarren et al. 2020; Lentz et al. 2020; Rael et al. 2022; Mitchell & Sullivan 2015; Sha et al. 2022; den Daas et al. 2020; John et al. 2020; John et al. 2020; Lentz et al. 2022; Okoboi et al. 2019; Rael et al. 2020; |
| 4 | General population: SNA was acceptable, especially when distributing HIVST kits, as it could provide earlier HIV identification, was convenient, avoided STI clinic-related stigma, and was empowering, but some raised concerns about the accuracy and legitimacy of the kit, and not receiving HIV results from a doctor. | Moderate confidence | Minor concerns regarding methodological limitations, No/Very minor concerns regarding coherence, Minor concerns regarding adequacy, and Moderate concerns regarding relevance | John 2018; Matovu et al. 2021; |
| 5 | General population: Men trusted their male peers more than their female partners whendiscussingt HIV testing since they have already discussed their sexual behaviours with each other. | Low confidence | No/Very minor concerns regarding methodological limitations, No/Very minor concerns regarding coherence, Serious concerns regarding adequacy, and No/Very minor concerns regarding relevance | Conserve et al. 2018; |
| 6 | FSW: The acceptability of SNA strategies varied according to the nature of their emotional or social relationship with their social network, with greater acceptability for their main partner. | Moderate confidence | Minor concerns regarding methodological limitations, Minor concerns regarding coherence, Minor concerns regarding adequacy, and No/Very minor concerns regarding relevance | Tucker JD et al. 2011; Ky- Zerbo O et al. 2022; Maman S et al. 2017; |
| 1. Social harms and adverse events | | | | |
| 7 | All populations: Fear of intimate partner violence, relationship dissolution, discrimination and stigma. | Low confidence | Minor concerns regarding methodological limitations, Serious concerns regarding coherence, Minor concerns regarding adequacy, and Minor concerns regarding relevance | John 2018; Lentz et al. 2020; Tembo et al. 2020; Conserve et al. 2018; Lentz et al. 2022; Okoboi et al. 2019; Rael et al. 2020; Tobin et al. 2018; Wango et al. 2021; |
| 8 | All populations: Bringing HIVST kits into a relationship or an invitation to test may be perceived as a sign of mistrust. | High confidence | No/Very minor concerns regarding methodological limitations, No/Very minor concerns regarding coherence, Minor concerns regarding adequacy, and No/Very minor concerns regarding relevance | John 2018; John et al. 2020; |
| 1. Barriers and facilitators | | | | |
| 9 | Adolescents and young women: Facilitators of SNA included training to support communication with their male partners, waiting for a time that a partner was in the right mood or temperament, and through positive messaging around utilising testing to strengthen their relationship | Moderate confidence | No/Very minor concerns regarding methodological limitations, No/Very minor concerns regarding coherence, Moderate concerns regarding adequacy, and Minor concerns regarding relevance | Tembo et al. 2020; Wango et al. 2021; |
| 10 | Adolescents and young women: Men emphasised that they would not want to feel ambushed and would want to have a sense of what would happen if they tested positive (specifically, men wanted assurances that if they tested positive, their relationship would not end). | Moderate confidence | No/Very minor concerns regarding methodological limitations, No/Very minor concerns regarding coherence, Moderate concerns regarding adequacy (due to reduced quantity and richness of data), and No/Very minor concerns regarding relevance | Tembo et al. 2020; Wango et al. 2021; |
| 11 | Youth: Facilitators of SNA included financial incentives, privacy and convenience of using HIVST, but some were wary of receiving health information from friends perceived as non-professionals while others avoided sharing personal issues with peer navigators from their community. | Moderate confidence | No/Very minor concerns regarding methodological limitations, No/Very minor concerns regarding coherence, Minor concerns regarding adequacy, and No/Very minor concerns regarding relevance | Adeagbo et al. 2022; Wango et al. 2021; |
| 12 | MSM/TGW: Facilitators of SNA included having trust between distributor and recipient, use of free or subsidised HIVST kits, having external support, and the strategy of broaching the topic, i.e. framing it as a component of research study, as a way of joint testing, being straightforward, as a prerequisite of having sex. | High confidence | No/Very minor concerns regarding methodological limitations, No/Very minor concerns regarding coherence, No/Very minor concerns regarding adequacy, and No/Very minor concerns regarding relevance | Frasca et al. 2014; Iribarren et al. 2020; Mitchell & Sullivan 2015; Sha et al. 2022; den Daas et al. 2020; John et al. 2020; Lentz et al. 2022; Tobin et al. 2018; |
| 13 | MSM/TGW: Barriers of SNA included concerns about lack of support for linkage to care, disclosure about self or others' sexual behaviour, the feeling of awkwardness, forgetting/getting caught up in the moment, loss of passion in sex when the topic was broached, the uncertainty of partners' reaction, worries about stigma and perception of infidelity, vulnerable conversation about sexual health, as well as diiculty contacting partners. | High confidence | No/Very minor concerns regarding methodological limitations, No/Very minor concerns regarding coherence, No/Very minor concerns regarding adequacy, and No/Very minor concerns regarding relevance | Frasca et al. 2014; Iribarren et al. 2020; Lentz et al. 2020; Mitchell & Sullivan 2015; Sha et al. 2022; den Daas et al. 2020; John et al. 2020; Lentz et al. 2022; Tobin et al. 2018; |
| 14 | General population: Facilitators include pre-SNA counselling session to prepare them to facilitate SNA and avoid possible violence. | Moderate confidence | No/Very minor concerns regarding methodological limitations, No/Very minor concerns regarding coherence, Moderate concerns regarding adequacy (given the reduced quantity and richness of data), and No/Very minor concerns regarding relevance | John 2018; Matovu et al. 2021; |
| 15 | General population: Barriers to SNA including worries about their partnersʼ reaction (including the potential for violence), lack of support when using HIVST kits (including for illiterate members), and diiculties locating social network members. | Moderate confidence | No/Very minor concerns regarding methodological limitations, No/Very minor concerns regarding coherence, Moderate concerns regarding adequacy (reduced quantity and richness of data), and No/Very minor concerns regarding relevance | John 2018; Matovu et al. 2021; |
| 16 | General population: For men, using formal and informal conversations facilitated SNA, as well as accompanying friends to the clinic as a means of social support and assurance, but some men expressed concerns about lack of confidentiality and privacy associated with attending local clinic, which may be staffed by people from the same neighbourhood, and can lead to stigmatisation if one is diagnosed with HIV. | Moderate confidence | No/Very minor concerns regarding methodological limitations, No/Very minor concerns regarding coherence, Moderate concerns regarding adequacy, and No/Very minor concerns regarding relevance | Conserve et al. 2018; |
| 17 | FSW: Facilitators for SNA include sharing positive experiences about testing (informational support), directly delivering HIVST kits (instrumental support and provides confidentiality), and encouraging linkage to care (emotional support). | Moderate confidence | Minor concerns regarding methodological limitations, No/Very minor concerns regarding coherence, Moderate concerns regarding adequacy due to lack of quantity and richness of data, and No/Very minor concerns regarding relevance | Ky-Zerbo O et al. 2022; McGowan M et al. 2022; |
| 18 | FSW: Barriers of SNA included the spread of misinformation about HIV and HIVST, fear of social exclusion following HIV status disclosure. | Moderate confidence | Minor concerns regarding methodological limitations, No/Very minor concerns regarding coherence, Moderate concerns regarding adequacy due to only 1 study contributing to this finding, and No/Very minor concerns regarding relevance | McGowan M et al. 2022; |
